# TERRA and the histone methyltransferase Dot1 cooperate to regulate senescence in budding yeast

**DOI:** 10.1371/journal.pone.0195698

**Published:** 2018-04-12

**Authors:** Jennifer J. Wanat, Glennis A. Logsdon, Jordan H. Driskill, Zhong Deng, Paul M. Lieberman, F. Brad Johnson

**Affiliations:** 1 University of Pennsylvania School of Medicine, Pathology and Laboratory Medicine, Philadelphia, Pennsylvania, United States of America; 2 Washington College, Department of Biology, Chestertown, Maryland, United States of America; 3 Department of Biochemistry and Biophysics, Perelman School of Medicine, University of Pennsylvania, Philadelphia, Pennsylvania, United States of America; 4 Department of Physiology, University of Texas Southwestern Medical Center, Dallas, Texas, United States of America; 5 The Wistar Institute, Gene Expression and Regulation, Philadelphia, Pennsylvania, United States of America; University of North Carolina at Chapel Hill, UNITED STATES

## Abstract

The events underlying senescence induced by critical telomere shortening are not fully understood. Here we provide evidence that TERRA, a non-coding RNA transcribed from subtelomeres, contributes to senescence in yeast lacking telomerase (*tlc1Δ*). Levels of TERRA expressed from multiple telomere ends appear elevated at senescence, and expression of an artificial RNA complementary to TERRA (anti-TERRA) binds TERRA *in vivo* and delays senescence. Anti-TERRA acts independently from several other mechanisms known to delay senescence, including those elicited by deletions of *EXO1*, *TEL1*, *SAS2*, and genes encoding RNase H enzymes. Further, it acts independently of the senescence delay provided by *RAD52*-dependent recombination. However, anti-TERRA delays senescence in a fashion epistatic to inactivation of the conserved histone methyltransferase Dot1. Dot1 associates with TERRA, and anti-TERRA disrupts this interaction *in vitro* and *in vivo*. Surprisingly, the anti-TERRA delay is independent of the C-terminal methyltransferase domain of Dot1 and instead requires only its N-terminus, which was previously found to facilitate release of telomeres from the nuclear periphery. Together, these data suggest that TERRA and Dot1 cooperate to drive senescence.

## Introduction

Telomeres shorten with DNA replication because of the end replication problem and other factors such as oxidative damage, exonucleolytic processing, and aberrant replication and recombination events [1]. Telomerase can counter this shortening but its level in most human tissues is not sufficient to compensate for the loss of length with age. As a result, telomeres shorten to critical lengths that can lead to apoptosis or permanent cell cycle arrest (senescence), depending on cell context [2]. In humans, there is increasing evidence that telomere shortening impairs tissue function and contributes to age-related diseases [3–5]. Unlike humans, the budding yeast *Saccharomyces cerevisiae* expresses telomerase in an effectively constitutive fashion, and therefore, telomere length is constantly maintained. However, deletion of genes encoding telomerase components, such as the RNA template, *TLC1*, or the catalytic subunit, *EST2*, causes telomeres to shorten with replication until they become critically short and the cells senesce [6,7]. Thus, this yeast model system, unlike the yeast chronological and mother cell replicative aging models, allows the study of senescence caused specifically by telomere shortening. Although most yeast cells senesce in the absence of telomerase, rare “survivors” can bypass senescence by utilizing homologous recombination (HR) pathways requiring the protein *RAD52* [8,9], similar to ALT cells in humans.

In yeast, all chromosome termini consist of subtelomeric X-elements and some also contain 1–4 tandem Y’-elements. These are followed by about 350 bp of an imperfectly repeated TG_1-3_ sequence that ends in a 3’ single stranded overhang of 13–15 nt [10–12]. The telomere end is bound by protein complexes, which include the Cdc13/Stn1/Ten1 and yKu70/80 complexes that create a “cap” [13,14]. This cap aids in genome stability by preventing activation of DNA damage checkpoints, chromosome recombination events that may result in deleterious genome rearrangements, and chromosome degradation by exonucleases such as Exo1 [15,16].

Telomeres were long thought to be transcriptionally repressed. However, telomeres from yeast to humans are now known to encode RNAs called Telomere Repeat-Containing RNA (TERRA; [17–19]). Transcription of TERRA begins in the subtelomere, using the C-rich strand as a template to encode G-rich transcripts, and can extend as far as two-thirds the length of the TG_1-3_-repeats [18]. Thus, each telomere’s TERRA molecule contains a unique subtelomeric sequence followed by telomeric repeat sequences. These RNAs, most often transcribed by RNA polymerase II, can vary in size from approximately 100–1200 bp in yeast and can be up to 9 kb long in humans [17–19]. In yeast, additional molecules consisting of only subtelomeric sequences (called ARRET) are transcribed in the orientation opposite to TERRA [18]. Finally, although not naturally present in budding yeast, C-rich telomere repeat-containing RNAs can be detected in fission yeast, mouse, humans, and plants, and are similar to the artificial anti-TERRA RNA we describe below [17–21].

TERRA levels have been found to correlate with telomere length [22,23] and telomeric damage [24–27]. The mammalian histone methyltransferase MLL interacts with p53 to increase transcription of TERRA at uncapped telomeres and to induce senescence [24]. Furthermore, telomere loss is correlated with increased TERRA levels in ICF (Immunodeficiency, Centromeric region instability, Facial anomalies) patients [25,28]. Together, these data suggest a potential role for TERRA in signaling telomere dysfunction.

Here we provide evidence that TERRA plays a role in signaling cellular senescence caused by critically shortened telomeres. We demonstrate that expression of an artificial anti-TERRA C_1-3_A-repeat RNA can delay senescence, and that this delay is associated with an unanticipated interplay between TERRA and the Dot1 protein. Dot1 interacts physically with TERRA, and anti-TERRA expression prevents this association. Together with our observation that genetic inactivation of Dot1 delays senescence in a fashion epistatic to anti-TERRA, our findings indicate that TERRA and Dot1 cooperate to drive senescence.

## Materials and methods

### Yeast strains and plasmids

All experiments were performed in the BY4741/4742 background. Deletions were of the entire open reading frame and were made by standard PCR-based disruption and transformation techniques or by crossing to the yeast deletion collection [29]. All disruptions were confirmed by PCR and auxotrophy or drug resistance. *TLC1/tlc1Δ* strains made by crossing were restruck at least 3X to equilibrate telomere lengths before use. See S1 Table for strain list.

To create pGL2, an ARS/CEN plasmid from which anti-TERRA was expressed from the *GAL1* promoter, a 277 bp region from plasmid pBJ1313 [30] that contains a cloned 262 bp *S*. *cerevisiae* telomere 1L repeat (TGTGTGTGTGTGGGTGTGGTGTGGTGTGTGGTGTGGTGTGTGGGTGTGTGGGTGTGGTGTGTGTGGTGTGTGGGTGTGGGTGTGGTGTGGGTGTGGGTGTGGTGTGTGGGTGTGGGTGTGGTGTGGGTGTGGGTGTGGTGTGTGGGTGTGGTGTGGGTGTGGTGTGGGTGTGGTGTGGTGTGTGGGTGTGGTGTGGGTGTGGGTGTGTGGGTGTGGGTGTGGTGTGTGTGGGTGTGGTGTGGGTGTGGTGTGGGTGTGTGTG) was PCR amplified, cut with SpeI and XhoI, and inserted into the same sites of pSH62 [31], replacing its cre-encoding sequences. For the control plasmid pGL3, pSH62 was digested with ClaI and religated to remove the cre-encoding sequence, thus enabling the *GAL1* promoter to express an effectively random sequence of 582 bp. To create pJD1, a plasmid encoding a 5’ MS2 tagged anti-TERRA transcript, a 62 bp sequence containing a 43 bp MS2 tag (CGTACACCATCAGGGTACGTTTTTCAGACACCATCAGGGTCTG) flanked by SpeI sites was cut and inserted into the SpeI site of pGL2. Plasmids pFvL901, 905, and 914 were a gift of Fred VanLeeuwen [32]. Plasmid pHMM (for MBP-MS2 purification) was a gift from Jeffrey S. Kieft [33]. The identities of all plasmids, including newly cloned sequences were confirmed.

### Yeast harvests

Unless stated otherwise, cells were grown to mid-to-late-log phase, spun down for 3 minutes at 1500 g, 4°C, washed 1X with ice-cold PBS or sterile water, flash frozen in liquid nitrogen, and stored at -80°C until use. All strains grew for at least 4 PD in fresh medium before harvesting. Senescent cultures were harvested about 5 PD before the low point of growth to minimize any contribution from survivors.

### Senescence assays

Heterozygous diploid *TLC1/tlc1Δ* strains were sporulated and haploid progeny were dissected, grown on YPAD and genotyped. Cells at ~PD = 25 after spore germination were resuspended in 7 mL of selective synthetic complete (SC) liquid media (SC-HIS for pGL2, pGL3 or pGJD1, and SC-HIS-LEU when also using the pFvL plasmids) with 2% raffinose as the carbon source and grown for 14–22 hours. Cells were then counted with a Coulter counter and 1.4x10^6^ cells were transferred into two new tubes with 7 mL of either 2% galactose (induced) or 2% glucose (uninduced) as the carbon source. Every 22 hours these cultures were counted and rediluted as above. The cell count was also used to calculate the number of PDs for each individual culture. In some cases, as indicated, senescence assays were initiated using colonies from germinated spores inoculated directly into selective SC medium containing glucose or galactose, rather than following initial growth in raffinose. All p values are two-tailed t-tests comparing the PD at the lowest points of growth (senescence) for individual cultures in the genotypic or treatment groups being compared. When comparisons were made between uninduced and induced samples of the same genotype, paired t-tests were performed, otherwise, tests were unpaired. For unpaired tests, F-tests were also performed; if the variance between samples was significantly different, t-tests assuming unequal variances were used instead. All statistics were performed using the VassarStats website.

### Telomere length measurements

Southern analysis was performed and quantified as described in Kozak et al. [34].

### MBP-MS2 pulldown assays

All steps were performed with ice-cold materials, and all washing and binding steps were performed with rotation unless otherwise stated. All washes are for 5 minutes. Yeast pellets from 10 mL of culture were resuspended in 500 μL lysis buffer (50 mM NaPO_4_ buffer pH 8.0, 140 mM NaCl, 1 mM EDTA pH 8.0, 1% Triton X-100, 0.05% Tween 20, 10 mM imidazole, 0.284 ng/μL leupeptin, 1.37 ng/μL pepstatin A, 0.33 ng/μL benzamidine, 8.5 ng/μL PMSF, 0.1mg/mL *E*. *coli* tRNA, 40 U RNasin/mL (Promega)) per pellet and disrupted using a mini-bead beater (6X 1 minute beat, 2 minute cooling) and zirconia/silica beads. Cells were washed from the beads with an additional 200 μL lysis buffer. Cleared lysate equivalent to 1x10^8^ cells for the pulldowns and 1x10^6^ cells for the inputs were treated with DNase I (Amplification Grade DNase I, Sigma) by adding 1X Reaction buffer (included with the Amplification Grade DNase I), 3 μL RNasin and 24 μL DNase I to each sample, and incubating at 37°C for 20 minutes. Reactions were stopped by adding 1X Stop Solution (included with the Amplification Grade DNase I) and incubating for 2 minutes at room temperature with occasional agitation. Samples were then centrifuged at max speed for 10 minutes, 4°C and supernatants were transferred to a new microcentrifuge tube. Input sample volumes were adjusted to 150 μL with elution buffer (50 mM NaPO_4_ buffer pH 8.0, 300 mM NaCl, 0.5% Tween 20, 250 mM imidazole, 40U/mL RNasin (Promega)). 0.5 mg of 6X-His-tagged-MBP-MS2 protein, purified essentially as described in [33], was prebound for 2 hours to 50 μL Ni-NTA magnetic agarose beads (Qiagen) in 500 μL binding buffer (50 mM NaPO_4_ buffer pH 8.0, 300 mM NaCl, 0.05% Tween 20, 20 mM imidazole). Beads were then washed 2X with binding buffer and 2X with lysis buffer and incubated overnight with the DNase I-treated uninduced or induced MS2-tagged lysates. Beads were then washed 2X with lysis buffer and 4X with wash buffer (50 mM HEPES-KOH buffer pH 7.5, 150 mM NaCl, 10 mM MgCl_2_, 0.05% Tween 20, 10 mM imidazole). MBP-MS2-RNA complexes were eluted 2X with 150 μL elution buffer for 10 minutes and pooled. Input and pulldown samples were treated with 20 μg proteinase K for 1 hour at 42°C and RNA was purified using MaxExtract tubes (Qiagen) as described by the manufacturer with an equal volume of acid phenol: chloroform: isoamyl alcohol (25:24:1, pH 4.5, Ambion) and precipitated overnight at -80°C with 20 μg glycogen (Thermo, RNA grade), 1/10^th^ volume 3M sodium acetate, and 2.5X volume 100% ethanol. Pellets were washed with 70% ethanol, dried, and resuspended in 90 μL DEPC-treated water (Ambion) at room temperature. Samples were DNase I treated with the TURBO DNase kit (Ambion) as described by the manufacturer, treated with RNase-free DNase I (Qiagen) in solution as described in the Qiagen RNeasy Mini Kit, and eluting 2X with 30 μL DEPC-treated water. Samples were then processed as described for quantitative real time PCR.

### TERRA-Dot1 binding assays

Yeast WCEs were prepared as described above for MBP-MS2 pulldowns. Yeast nuclear extracts were prepared by differential centrifugation as described in Dunn and Wobbe [35] except that the Ficoll buffer was modified to contain 1.5 mM EDTA, and instead of the 1X protease inhibitor mix, contained 0.2% fungal protease inhibitors (Sigma P8215), 0.2 mg/mL benzamidine, 1 μg/mL leupeptin, and 1 μg/mL pepstatin A; the extraction buffer was modified to contain 0.4 M ammonium sulfate, 2.5 mM EDTA, 20 mM potassium acetate 1% fungal protease inhibitors, 0.1 mg/mL benzamidine, 10 μg/mL leupeptin, and 10 μg/mL pepstatin A; and the lysates were centrifuged for 80 minutes at 100,000 g in a TLA 120.3 rotor. All steps were performed on ice or at 4°C.

RNA affinity purification was performed as described previously [26] with minor modifications. Briefly, biotinylated RNA probes included the following sequences: yeast TERRA, 5’-/Bio/-UGGGUGUGGUGUGGGUGUGGUGUGGGUGUGGUG; yeast anti-TERRA, 5’-/Bio/- CACCACACCCACACCACACCCACACCACACCCA; and control 5’-/Bio/-(CACUGA)6. Biotinylated RNA oligos (~2 nmol) were coupled to Dynabeads M-280 Streptavidin (Life Technologies) using DEPC-treated water prepared 2X B&W buffer (10 mM Tris pH 7.5, 2 M NaCl, 1 mM EDTA), essentially as described by the manufacturer’s instruction. Yeast cell extracts (1x10^9^ cells) diluted in D150 buffer (20 mM HEPES pH 7.9, 20% glycerol, 0.2 mM EDTA, 150 mM NaCl, 0.05% NP-40, 1 mM PMSF, 10 mM 2-mercaptoethanol) supplemented with yeast protease inhibitor cocktail (Sigma P8215) and 50 U/mL SUPERasedin (Ambion) were pre-cleared with control (CACUGA)6 RNA-coupled streptavidin beads twice for 30 minutes each with rotation at 4°C. The cleared nuclear extracts were further incubated with yeast TERRA or anti-TERRA RNA-coupled streptavidin beads for 1 hour with rotation at 4°C. The bound materials were washed five times with D150 buffer, and eluted with 100 μL of 1X B&W buffer (5 mM Tris pH 7.5, 1 M NaCl, 0.5 mM EDTA) for 15 minutes at 4°C. The elutes were concentrated by TCA precipitation and the remaining species on the beads were boiled in 2X Laemmli buffer prior to SDS-PAGE, western blotting, or LC/MS/MS analysis.

RNA affinity pulldown of V5-tagged Dot1 was performed as described for RNA affinity purification with some modifications. Briefly, yeast nuclear extracts isolated from V5-Dot1 expressing cells (2x10^8^ cells) were diluted three-fold in D150 buffer and used for binding to yeast TERRA-, anti-TERRA-, or control RNA-coupled streptavidin beads separately for 1 hour with rotation at 4°C. The beads were washed 3X with D150 buffer, 2X with D250 buffer, and boiled in 2X Laemmli buffer for SDS-PAGE and western blotting analysis. For binding assays with the TERRA/anti-TERRA duplex, equal amount of each RNA oligo (2nmol) was annealed in anneal buffer (10mM Tris-HCl pH 8.0 containing either 20 mM NaCl or 20 mM LiCl) by heating at 94°C for 1 minute and cooling slowly to room temperature. The resultant RNA duplex was used for coupling to Dynabeads and affinity pulldown assays.

### Western blotting

Membranes were blocked in TBS-T (20 mM Tris-HCl pH 7.5, 200 mM NaCl, 0.02% Tween 20) with 5% milk and all washing steps were done in TBS-T. Membranes were probed with 1:1000 rabbit anti-Dot1 (gift of VanLeeuwen lab) in 1% milk and TBS-T, 1:5000 mouse anti-V5 (Invitrogen, 46–0705) in 2% milk and TBS-T or 1:1000 mouse anti-β-actin (Abcam, 8224) in 1% milk and TBS-T followed by 1:5000 HRP conjugated goat anti-rabbit IgG (H+L) secondary (BioRad, 170–6515) or 1:10,000 HRP conjugated goat anti-mouse IgG (H+L) secondary (Jackson, 115-035-146). Blots were imaged using SuperSignal West Pico or Femto Chemiluminescent (Thermo) and a BioRad ChemiDoc XRS+.

### Chromatin immunoprecipitation

Cells were crosslinked with 1% formaldehyde in culture medium for 10 minutes shaking at room temperature. Crosslinking was stopped by adding a final concentration of 125 mM glycine and incubating at room temperature for 5 minutes. All IP steps were performed at 4°C and washes and binding steps were performed with rotation. All washes were 5 minutes. Cell pellets were disrupted in FA-Lysis buffer (50 mM Hepes-KOH 7.5, 140 mM NaCl, 1 mM EDTA, 1% TritonX-100, 0.1% sodium deoxycholate, 0.284 ng/μL leupeptin, 1.37 ng/μL pepstatin A, 0.33 ng/μL benzamidine, 8.5 ng/μL PMSF) as described for the MBP-MS2 pulldown assays. The lysate was sonicated to an average size of 100–500 bp as described in Kozak et al. [34]. 30μL of Protein A Dynabeads were washed 3X in block solution (5 mg/mL BSA in PBS), and 4 μg of rabbit anti-total Histone H3 (Abcam, ab1791), anti-H3K79me3 (Abcam, ab2621), or with total rabbit IgG (Pierce, 31207) were prebound for 6 hours. Bead-antibody complexes were then washed 2X with block solution and 2X with FA-lysis buffer. 750 μg total pre-cleared lysate (measured by Bradford assay) was used for IPs and 7.5 μg was used for inputs. IPs were bound to beads overnight. Bead-antibody-protein complexes were washed 2X with FA-lysis buffer, 2X with FA-lysis 500 buffer (FA-lysis with 500 mM total NaCl), 2X LiCl solution (10 mM Tris-HCl pH 8.0, 0.25 M LiCl, 1 mM EDTA, pH 8.0, 0.5% Igepal CA-630, 0.1% sodium deoxycholate) and 1X TE + 0.1% Igepal CA-630. Elutions were performed 3X with 100 μL TES (50 mM Tris-HCl, pH 8.0, 10 mM EDTA pH 8.0, 1% SDS) at 65°C using a Thermomixer. Inputs and IPs were decrosslinked overnight with addition of 200 mM NaCl and incubation at 65°C, treated with 120 μg RNase A and then100 μg proteinase K each at 37°C for 1 hour, purified using the Qiagen PCR cleanup kit, and eluted 3X 80 μL EB. Quantification was performed as described in Platt et al. [36].

### Immunoprecipitation of V5-Dot1 and associated RNAs

All washes and binding steps were performed as described in the MBP-MS2 pulldowns. Yeast pellets were resuspended in 750 μL RIP lysis buffer (50 mM HEPES-KOH pH 7.5, 140 mM NaCl, 1 mM EDTA pH 8.0, 0.1% Triton X-100, 0.5% Igepal CA-630, protease inhibitors (100 μg/mL each leupeptin, pepstatin, and benzamidine; 1 μM PMSF), 1 μM DTT, 40 U/μL RNasin (Promega)) per 40 mL of culture and disrupted using a mini-bead beater (6X 1 minute beat, 2 minute cooling) and zirconia/silica beads (BioSpec). Cells were then washed from the beads with an additional 200 μL RIP lysis buffer. 10 μL of anti-V5 antibody (Invitrogen) was prebound overnight to 30 μL Protein G Agarose beads (Roche) in 300 μL RIP lysis buffer. Beads were then washed 3X with RIP lysis buffer and incubated for 3 hours with tagged or untagged cleared lysate equivalent to 4x10^8^ cells. Protein inputs (1% of total) were flash frozen in liquid nitrogen for later use. RNA inputs (10% of total) were put on ice during the incubation and wash steps and then processed at the same time as the IPs. The beads were then washed 4X with RIP lysis buffer and resuspended in 300 μL RIP lysis buffer. 10% of the bead mixture was mixed with 2X SDS loading buffer as a protein IP control and flash frozen until processed. 90% of the bead mixture was extracted with an equal volume of acid phenol: chloroform: isoamyl alcohol (25:24:1, pH 4.5, Ambion) and precipitated overnight at -80°C with 20 μg glycogen (Thermo, RNA grade), 1/10^th^ volume 3M sodium acetate, and 2.5X volume 100% ethanol. Pellets were washed with 70% ethanol, dried, and resuspended in 87.5 μL DEPC-treated water (Ambion) at room temperature. Input samples were treated with RNase-Free DNase I using the RNeasy Mini kit (both Qiagen) in solution and 2X on column as described in the RNA preparation and quantitative real time PCR section, below. IPs were treated with DNase I in solution and 1X on column as described in the aforementioned kit eluting 2X 45 μL DEPC-treated water. All samples were then treated with DNase I in solution and cleanup was performed using the Qiagen MinElute Kit and eluted in 14 μL DEPC-treated water. Samples were then treated as described, below, for quantitative real time PCR.

### RNA preparation and quantitative real time PCR for TERRA quantification

RNA was extracted from cells using a standard hot-acid phenol extraction protocol [37]. As described in Iglesias et al. [38], we also found that three DNase I treatments were needed to efficiently remove telomeric DNA background. Using RNase-free DNase I and the RNeasy Mini kit (both Qiagen), we performed one treatment in solution, followed by one on column treatment as directed. Samples were then washed with RW1 and then 2X with RPE before a last on column treatment was performed as described. Samples were then cleaned-up as directed and eluted 2X with 30 μL with DEPC-treated water (Ambion).

For reverse transcription, 3 μg of DNase I treated RNA, 2 mM dNTP blend (GeneAmp, AB), and 6.5 μM random hexamers (GeneAmp, Invitrogen) in a 13 μL volume were heated to 90°C for 1 minute and cooled to 25°C in a PCR machine. 200 U SuperScript III reverse transcriptase, 5 mM DTT, 1X First Strand buffer (all Invitrogen), and 40 U RNasin Plus RNase Inhibitor (Promega) were added in a final volume of 20 μL. Samples were then incubated 5 minutes at 25°C, 1 hour at 55°C, and 15 minutes at 72°C. Controls were also set up as described but lacking SuperScript and random hexamer primers.

For qPCR the cDNA was diluted so that samples were within the linear range of the standard curves. Oligo sequences are listed in S2 Table. 10 μL SybrGreen JumpStart TaqReadyMix (Sigma) reactions were quantified on a LightCycler 480 (Roche) as follows: 10 minutes at 95°C, 50 cycles of 95°C for 15 seconds, 58°C for 10 seconds, 68°C for 1 minute, and a dissociation analysis at 95°C. PCRs were run in triplicate. Samples with multiple melt curves were analyzed further by gel electrophoresis. Those with multiple bands were excluded from the dataset. Samples were normalized to cDNA standard curves and the housekeeping gene *SPC42*, which was chosen because its mRNA level does not change in senescing yeast cells [39].

## Results

### Levels of transcripts initiating in subtelomeres increase as cells become senescent

**Fig 1:**
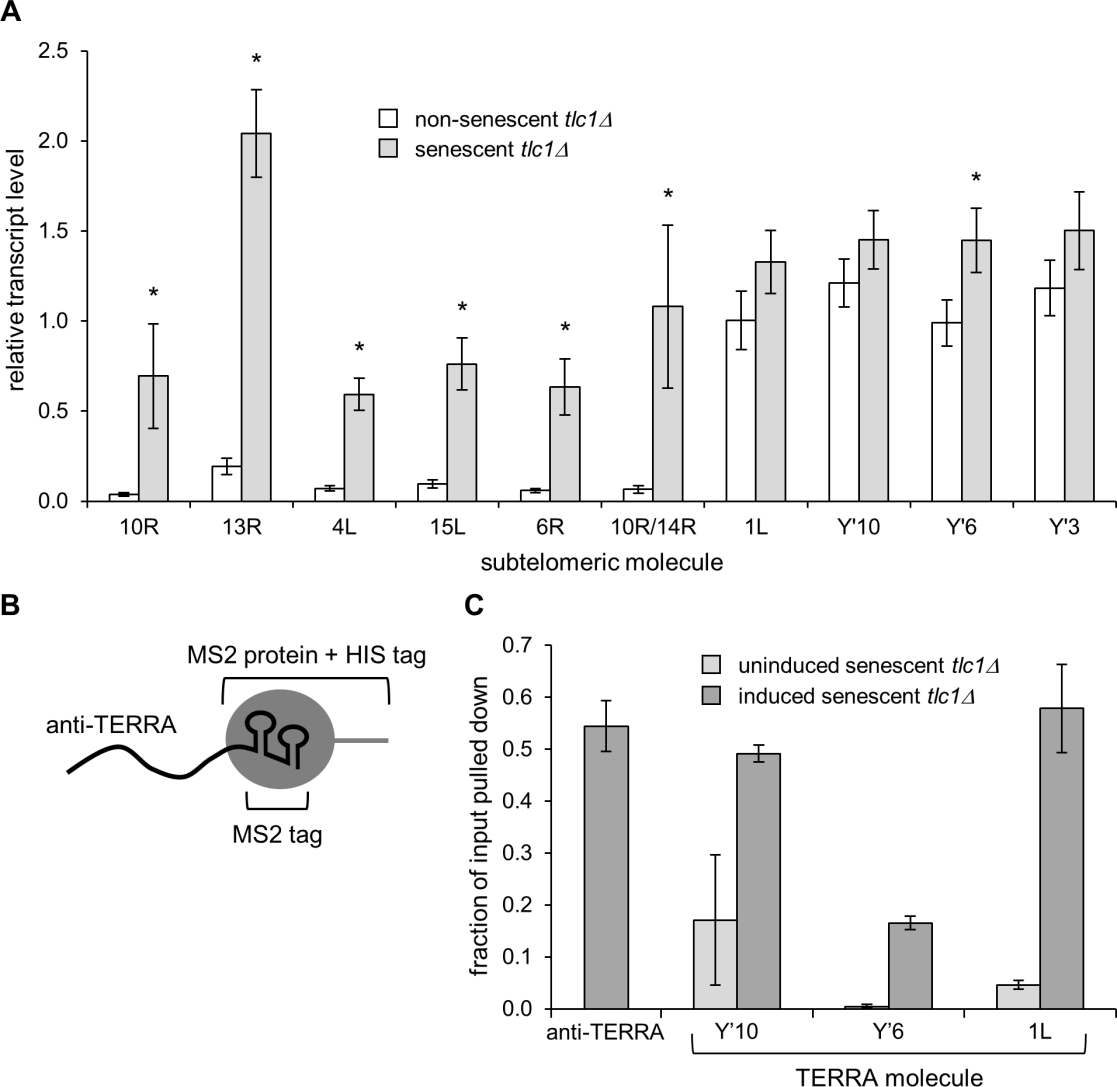
Anti-TERRA RNA binds TERRA *in vivo* in senescent *tlc1Δ* mutants. (A) Transcripts from multiple telomeres increase at senescence. Deletion of the telomerase RNA template, *TLC1*, causes telomeres to shorten until they undergo senescence. Transcript levels from the indicated telomeres of cells with longer telomeres (non-senescent *tlc1Δ*) versus cells with shorter telomeres (senescent *tlc1Δ*) were measured by qRT-PCR and normalized to housekeeping genes (see Materials and Methods). Error bars are the SEM (n = 9). p values ≤ 0.05 are indicated by an asterisk. (B) Anti-TERRA was fused to an MS2 RNA tag and was pulled down from senescent cell extracts using a 6X-His tagged MBP-MS2 coat fusion protein. (C) Anti-TERRA efficiently pulls down native TERRA *in vivo*. Bar graphs represent the average qPCR-based measurements of the fraction of total cellular anti-TERRA that is pulled down and the fractions of total cellular TERRA from particular telomeres that are pulled down along with anti-TERRA. n = 3 independent experiments. The fraction of anti-TERRA that is pulled down (~55%) is a control that reflects the maximum achievable efficiency of TERRA that could be pulled down along with anti-TERRA if all TERRA molecules are bound by anti-TERRA. The low levels of TERRA recovered in samples from cells in which anti-TERRA is not induced demonstrates the dependence of the TERRA detection on the expression of anti-TERRA.

Cellular senescence driven by telomere shortening can be modeled in *S*. *cerevisiae* by deletion of the telomerase RNA template, *TLC1*, causing telomeres to shorten with replication until they become critically short and the cells undergo an irreversible cell cycle arrest (senescence; [7]). As telomeres get shorter, the subtelomeric chromatin structure displays changes in post-translational histone modifications and protein associations characteristic of transcriptionally active chromatin ([34], and see below). Because of the aforementioned chromatin changes, and since TERRA is a set of non-coding RNA molecules transcribed from subtelomeric promoters and extending into the telomere repeats at chromosome ends [17–19], we hypothesized that TERRA levels might change and possibly play a functional role in triggering cellular senescence in yeast. qRT-PCR analyses of RNA from non-senescent *tlc1Δ* versus senescent *tlc1Δ* cells showed that levels of transcripts from multiple subtelomeres are indeed significantly increased at senescence (Fig 1A). In support of this finding, other groups recently also reported that TERRA levels are increased by telomere shortening [23,40].

### Anti-TERRA (C_1-3_A repeat) RNA binds TERRA *in vivo* and delays senescence in *tlc1Δ* mutants

Given the above results, we wanted to alter TERRA levels and examine the effect on senescence. However, because TERRA originates from multiple telomeres and is not known to be under the control of any dedicated regulator, there is no apparent way to selectively and comprehensively alter natural TERRA expression. Therefore, we created a tool, an artificial 262 nt C_1-3_A antisense-TERRA RNA, that could potentially interact with TERRA transcribed from any telomere in the yeast genome. The anti-TERRA RNA was fused to an MS2 RNA affinity tag and expressed from an ARS/CEN plasmid vector containing a cloned telomere repeat fragment whose transcription was driven by a galactose-inducible *GAL1* promoter. Using senescent cells in which anti-TERRA expression was induced, native TERRA molecules were pulled down in complex with anti-TERRA, in the absence of crosslinking, and nearly as efficiently as the anti-TERRA molecules themselves (Fig 1B and 1C), indicating that TERRA and anti-TERRA interact physically and quantitatively *in vivo*.

**Fig 2:**
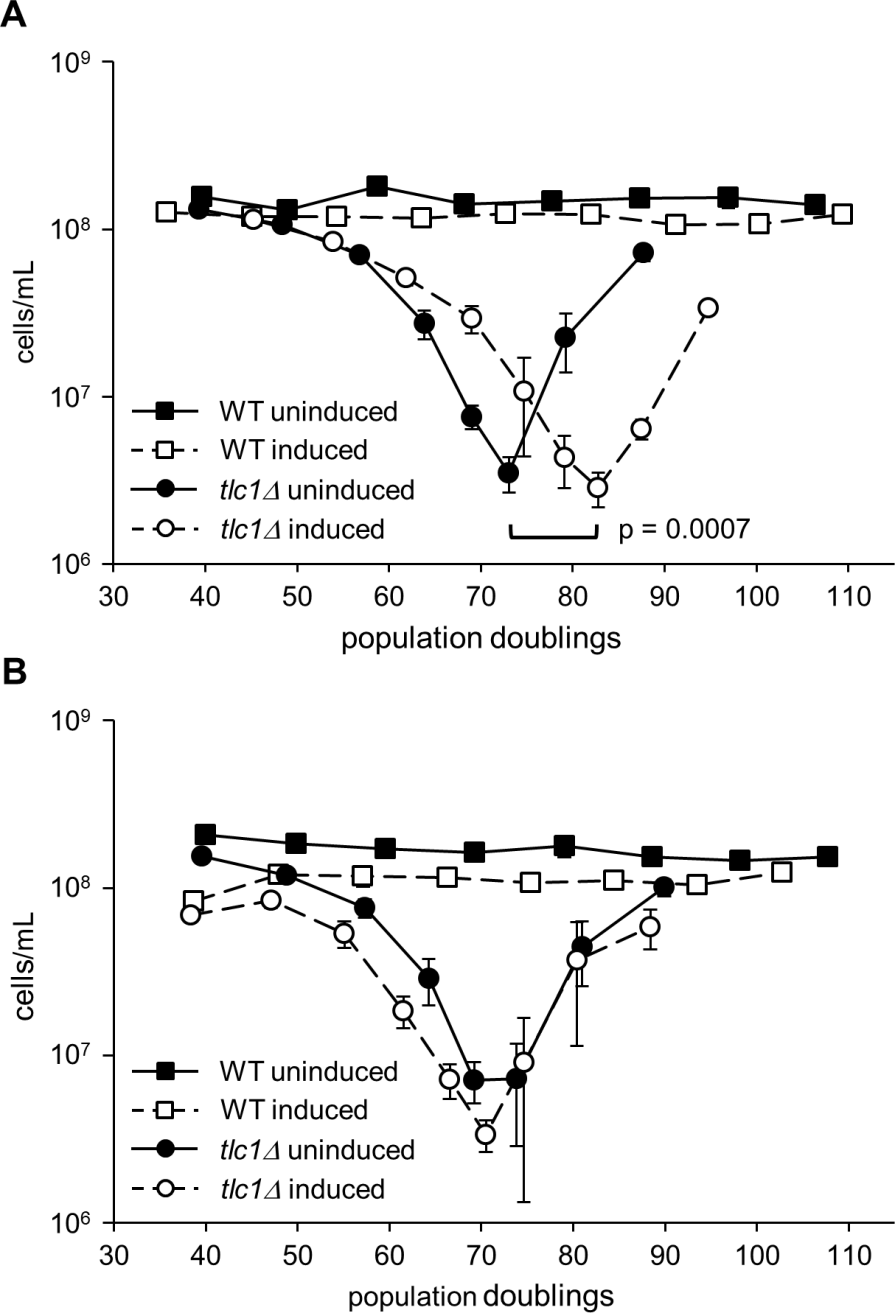
Anti-TERRA delays senescence in *tlc1Δ* mutants. (A) Anti-TERRA expression delays senescence in *tlc1* mutants by 10 PD (p = 0.0007). *TLC1/tlc1Δ* diploids carrying the inducible anti-TERRA plasmid were sporulated, and senescence assays of WT (n = 2) and *tlc1Δ* (n = 5) were performed as indicated in the *Materials and Methods* with anti-TERRA either induced or uninduced. (B) Induction alone does not delay senescence. *TLC1/tlc1Δ* diploids bearing an inducible control plasmid (which does not express anti-TERRA) were sporulated and senescence assays of WT (n = 2) and *tlc1Δ* (n = 5) were performed as indicated in the *Materials and Methods* with the control plasmid either induced or uninduced. In both panels, each data point represents the mean PD versus the mean and SEM of the cell density.

Since anti-TERRA interacts with TERRA, we reasoned that it might inhibit natural TERRA functions. We performed quantitative liquid senescence assays to determine whether anti-TERRA alters the rate of cellular senescence. The anti-TERRA vector was introduced into a *TLC1/tlc1Δ* diploid, which was sporulated to generate *tlc1Δ* haploid progeny bearing the vector, and senescence was monitored under uninduced versus induced conditions. As shown in Fig 2A, expression of anti-TERRA delayed senescence by approximately 10 population doublings (PDs, p = 0.0007) indicated by the shift of the curve to the right. Survivors account for the improved growth of cells after senescence [9,41] and are indicated by the sharp upswing of the curve after the growth nadir.

Control experiments using a vector transcribing a similarly-sized random DNA sequence confirmed that induced versus uninduced growth conditions by themselves do not affect senescence (Fig 2B) nor does addition of the MS2 RNA affinity tag (S1 Fig). Thus, anti-TERRA enables cells to undergo more cell doublings prior to senescence, rather than delaying senescence by simply slowing the rate of cell division. Anti-TERRA also delayed senescence in *est2Δ* strains (S2 Fig), ruling-out the possibility that in *tlc1Δ* strains anti-TERRA is acting as an alternative RNA template for the catalytic subunit of telomerase (Est2) to enable telomere lengthening (S3 Fig). Together, these data rule out the possibility that anti-TERRA delays senescence in a fashion specific only to deletion of *TLC1*.

### Anti-TERRA acts independently from several regulators of senescence and telomere capping

At least four types of genetic manipulations are known to delay cellular senescence: inhibition of DNA damage checkpoints, modification of telomeric chromatin, telomere RNA-DNA hybrid formation (i.e. R-loops), and interference with 5’ to 3’ exonucleolytic telomere resection. In contrast, *RAD52*, which is required for effectively all types of homologous recombination (HR), prevents premature senescence [9]. If anti-TERRA affects one of these mechanisms, the delayed senescence provided by anti-TERRA should be epistatic to that provided by alteration of the mechanism in question. Therefore, we tested whether anti-TERRA delayed senescence in cells also lacking each of the following: Tel1, Sas2, Rnh1 and Rnh201, Exo1, Rad9, or Rad52. Tel1 is a homologue of the ATM checkpoint kinase, it preferentially binds short telomeres [42,43] and its deletion delays senescence in a *tlc1Δ* background [34,44]. Sas2 is a histone acetyltransferase and its absence delays senescence in a *TEL1*-independent fashion [34]. Rnh1 and Rnh201 are required for the two yeast RNase H activities, and their combined absence promotes TERRA-telomere hybrids, telomere recombination, and delayed senescence [45,46]. Exo1 is the primary 5’ to 3’ exonuclease active at uncapped telomeres, and senescence is delayed in its absence [47,48]. Furthermore, TERRA can interfere with the ability of the Ku70/80 complex to protect telomere ends from Exo1 [49], raising the possibility that anti-TERRA inhibits Exo1. Lastly, the 53BP1 homolog Rad9 is not only important for checkpoint responses at uncapped telomeres [50–52] but also limits 5’ to 3’ end resection of uncapped telomeres by regulating the activity of Exo1 and another exonuclease activity termed ExoX [15,53].

**Fig 3:**
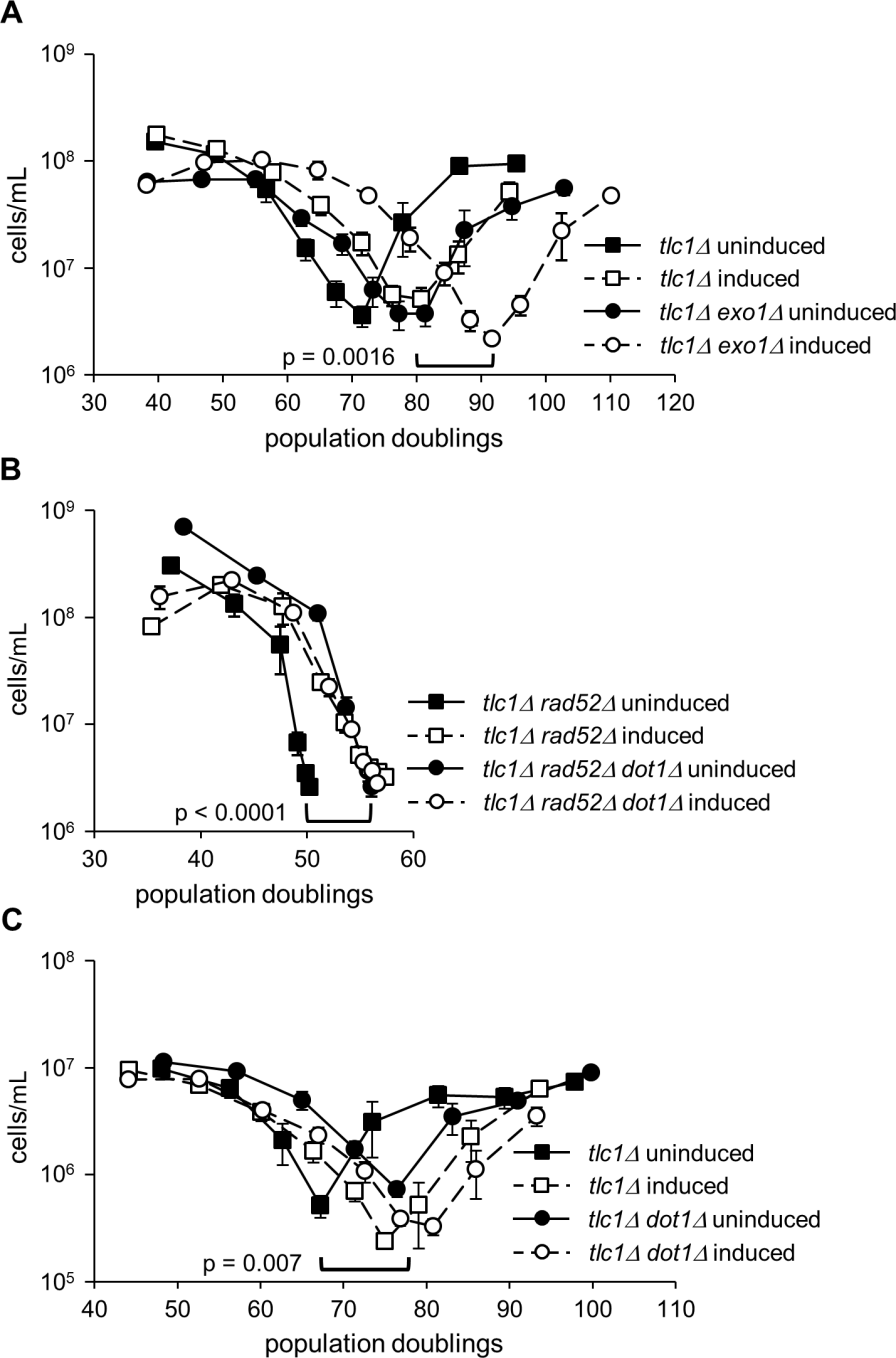
The anti-TERRA senescence delay is independent of Exo1 and Rad52 but is dependent on Dot1. (A) Anti-TERRA delays senescence in *tlc1Δ exo1Δ* mutants. *TLC1/tlc1Δ EXO1/exo1Δ* were sporulated and senescence assays of *tlc1Δ* and *tlc1Δ exo1Δ* (n = 5 each) were performed with anti-TERRA either induced or uninduced. Anti-TERRA and *exo1Δ* each significantly delay senescence (*tlc1Δ* uninduced versus *tlc1Δ* induced, 10 PD, p < 0.002 and *tlc1Δ* uninduced versus *tlc1Δ exo1Δ* uninduced, 9 PD, p = 0.001). Together they delay senescence even further (*tlc1Δ exo1Δ* uninduced versus *tlc1Δ exo1Δ* induced, 13 PD, p = 0.0016). (B) Anti-TERRA delays senescence in *tlc1Δ rad52Δ* mutants but not *tlc1Δ rad52Δ dot1Δ* mutants. *TLC1/tlc1Δ RAD52/rad52Δ DOT1/dot1Δ* diploids were sporulated and senescence assays of *tlc1Δ rad52Δ* (n = 5) and *tlc1Δ rad52Δ dot1Δ* (n = 6) were performed with anti-TERRA either induced or uninduced. Both anti-TERRA and *dot1Δ* delay senescence in the absence of Rad52 (*tlc1Δ rad52Δ* uninduced versus *tlc1Δ rad52Δ* induced, 10 PD, p < 0.0001 and *tlc1Δ rad52Δ* uninduced versus *tlc1Δ rad52Δ dot1Δ* uninduced, 9 PD, p = 0.005), but anti-TERRA does not cause a further delay in the absence of Dot1 (*tlc1Δ rad52Δ dot1Δ* uninduced versus *tlc1Δ rad52Δ dot1Δ* induced, p = 0.563). (C) Same as in (A) except that *dot1* deletion was tested instead of *exo1* deletion. Anti-TERRA and *dot1Δ* each delay senescence (*tlc1Δ* uninduced versus *tlc1Δ* induced, 8 PD, p = 0.002 and *tlc1Δ* uninduced versus *tlc1Δ dot1Δ* uninduced, 9 PD, p = 0.007). But again, anti-TERRA does not cause a further delay in the absence of Dot1 (*tlc1Δ dot1Δ* uninduced vs. *tlc1Δ dot1Δ* induced 1.5 PD, p = 0.238). In all panels, each data point represents the mean PD versus the mean and SEM of the cell density.

To address the potential involvement of anti-TERRA in the regulation of senescence by the factors just described, diploids heterozygous for deletion mutations in *TLC1*, heterozygous for deletions of the genes in question, and carrying the *GAL1*-driven anti-TERRA plasmid, were sporulated to generate haploids for senescence assays. This allowed for controlled comparisons in senescence because all haploid progeny came from the same epigenetic background and inherited telomeres of similar lengths. We found that anti-TERRA expression delayed senescence substantially even in the absence of Exo1, Tel1, Sas2, Rnh1 and Rnh201, or Rad9 (Fig 3A and S4 Fig), demonstrating non-epistatic relationships between anti-TERRA and the different factors.

We also explored potential roles for anti-TERRA in telomere capping using the temperature-sensitive *yku70Δ* and *yku80Δ* mutants [54]. These mutants suffer from 5’ to 3’ telomere end resection at elevated temperatures, conceivably similar to the degradation observed at critically shortened telomeres in telomerase-deficient cells [14,47,55]. However, anti-TERRA expression did not suppress the temperature sensitivity of either of these mutants (S5 Fig). These findings are consistent with the lack of a role for anti-TERRA in Exo1-dependent mechanisms during senescence (Fig 3A).

*RAD52*-dependent HR delays senescence in telomerase deficient cells [8,9,30,47]. However, anti-TERRA expression significantly delayed senescence of *tlc1Δ rad52Δ* mutants (Fig 3B, p < 0.0001). Therefore, anti-TERRA delays senescence in a fashion independent of HR. Altogether, these investigations indicate that anti-TERRA impacts a mechanism distinct from several established pathways of senescence regulation.

### Dot1 is required for the senescence delay caused by anti-TERRA

Dot1 is a highly conserved histone methyltransferase responsible for the mono-, di- and tri-methylation of histone H3 lysine 79 (H3K79) in organisms from yeast to humans [56–62]. Previous work from our laboratory has demonstrated that deletion of *DOT1* delays senescence [34]. We found that anti-TERRA does not add to the senescence delay provided by *dot1* deletion (Fig 3C) and *RAD52* is not required for this delay (Fig 3B). Therefore, anti-TERRA and *dot1Δ* both delay senescence as part of the same *RAD52*-independent pathway. Furthermore, the identical rates of senescence among *tlc1Δ dot1Δ rad52Δ* strains, regardless of the presence or absence of anti-TERRA induction, clearly demonstrates the epistatic relationship between anti-TERRA and *dot1* deletion.

These findings raise the possibility that TERRA drives senescence by interacting in some fashion with Dot1, and that anti-TERRA delays senescence by disrupting the TERRA-Dot1 interaction.

### Dot1 binds TERRA and this interaction is blocked by anti-TERRA

**Fig 4:**
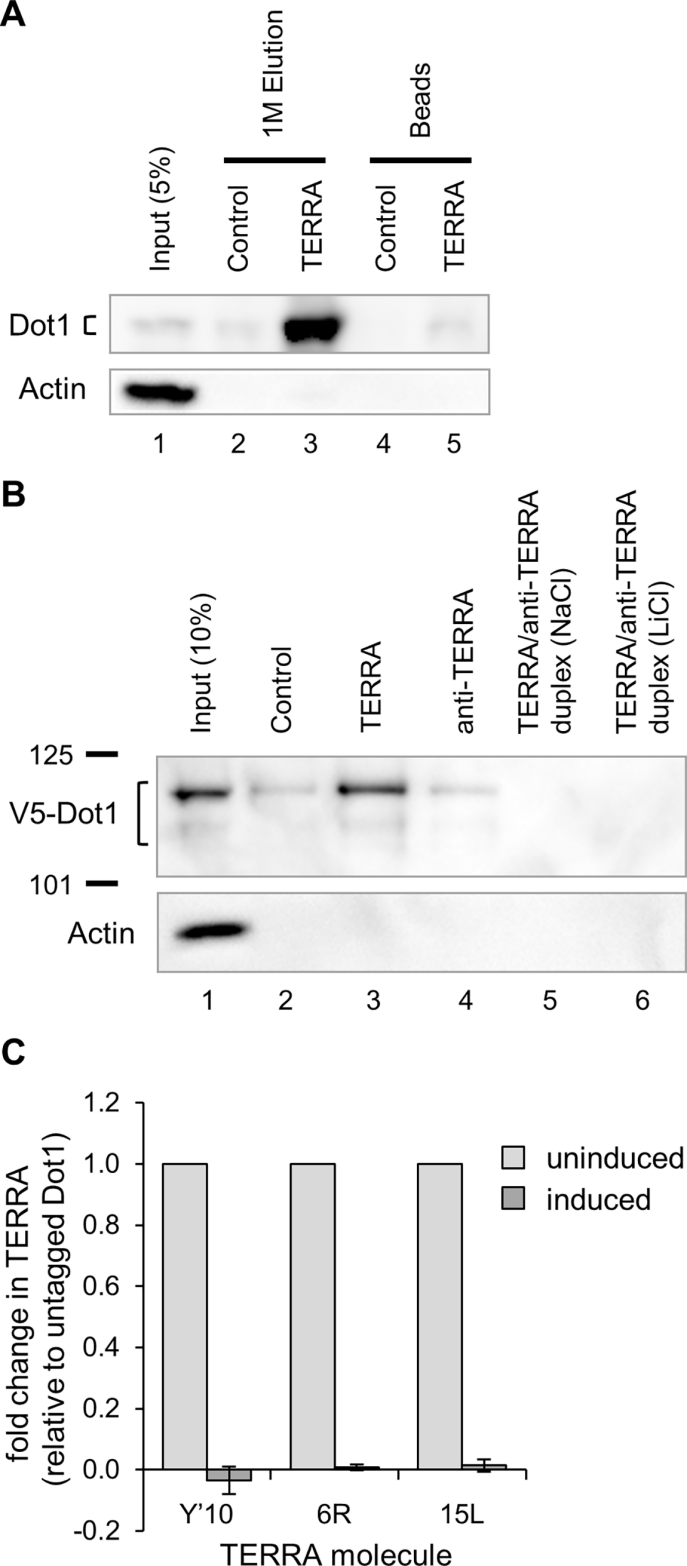
Dot1 associates with TERRA and anti-TERRA disrupts this interaction. (A) TERRA-like RNA oligonucleotides but not anti-TERRA molecules can pull down native Dot1 from yeast whole cell extracts. Yeast whole cell extracts (WCE) were subject to RNA affinity purification using biotinylated TERRA or control (random sequence) RNA oligonucleotides. Bound materials were eluted with 1M NaCl in lanes 2 and 3 and then the beads were boiled in lanes 4 and 5. Lane 1 is 5% of WCE as input. Proteins were visualized by western blot with anti-Dot1 antibody and anti- -actin antibody as a control. Dot1 protein migrates near 65 kD as a doublet. (B) V5-tagged Dot1 binds TERRA and anti-TERRA prevents this interaction *in vitro*. Nuclear extracts were subject to RNA pulldown using the indicated RNA templates. Bound materials were eluted with 2X Laemmli buffer by boiling and assayed by western blot with antibodies specific to V5 or Actin. Lanes 5 and 6: TERRA oligonucleotides were annealed to anti-TERRA molecules under G-quadruplex permissive or minimizing conditions (NaCl or LiCl, respectively) and then were then transferred in the standard buffer used for RNA pulldown. The LiCl conditions rule out the possibility that folding of TERRA into G-quadruplexes, rather than forming duplexes with anti-TERRA, explains the loss of Dot1 binding. Lane 1 is 10% of input. Marker size in kD are indicated at left. Both isoforms of Dot1 can be seen around 110 kD. (C) V5-tagged Dot1 binds TERRA and anti-TERRA prevents this interaction *in vivo*. RNA immunoprecipitation was performed with V5-tagged Dot1 on yeast WCE. TERRA levels are quantified by qRT-PCR and displayed as fold change relative to an untagged Dot1 strain and to input (n = 2).

To test if the Dot1 interaction with TERRA might involve direct (or indirect) binding between the two, we coupled biotinylated TERRA, anti-TERRA, and control RNA oligonucleotides to streptavidin beads, incubated them with yeast extracts, and assayed the bound proteins by western blot. Dot1 (untagged and V5-tagged) was highly enriched in eluates from TERRA but not control (Fig 4A and 4B) or anti-TERRA (Fig 4B) RNA beads, indicating that Dot1 associates specifically with TERRA RNA. We next asked whether anti-TERRA affects this interaction. Indeed, pre-assembly of the TERRA and anti-TERRA oligonucleotides into duplexes disrupted V5-Dot1 binding (Fig 4B, lanes 5 and 6).

To determine whether anti-TERRA impacts the association of Dot1 with TERRA *in vivo*, we performed RNA immunoprecipitation (RIP) under native conditions with V5-tagged Dot1 from cell extracts and measured enrichment of TERRA in the presence and absence of anti-TERRA expression. As expected, immunoprecipitation of V5-Dot1 also co-precipitated multiple TERRA species (Y’-TERRA, 6R-TERRA, and 15L-TERRA) but these interactions were lost when anti-TERRA was expressed (Fig 4C). Together, these results indicate that Dot1 interacts with TERRA *in vivo*, either directly or indirectly, and that anti-TERRA expression inhibits this interaction.

### Anti-TERRA delays senescence by blocking the N-terminal function, but not the C-terminal methylation activity, of Dot1

**Fig 5:**
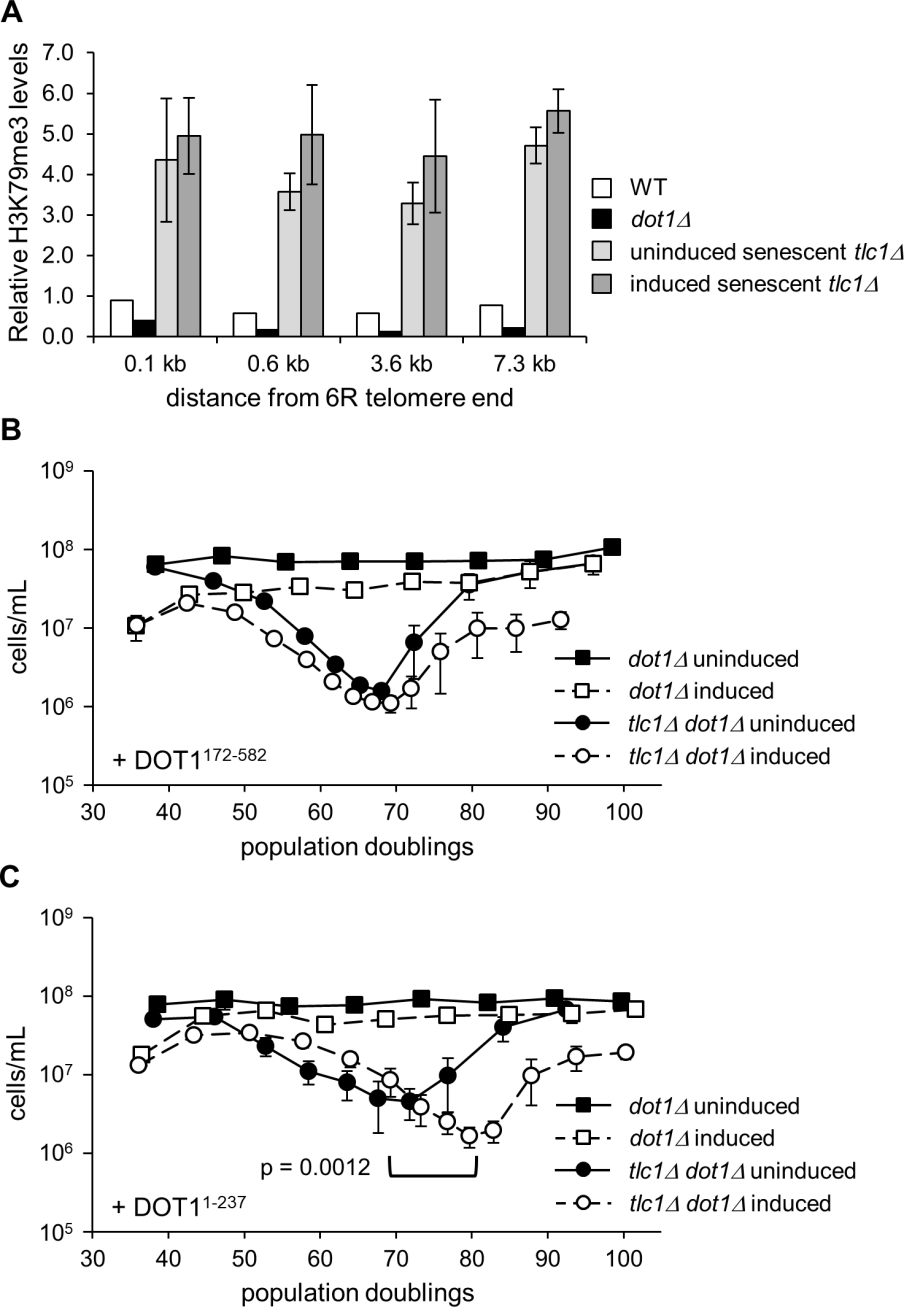
The N-terminus of Dot1 is necessary and sufficient for delay of senescence by anti-TERRA. (A) Anti-TERRA expression does not significantly alter subtelomeric H3K79me3 levels in senescent *tlc1Δ* cells. Chromatin immunoprecipitation (ChIP) was performed on the indicated strains with control IgG, H3K79me3, or histone H3 antibodies. H3K79me3 levels at the indicated regions of the 6R subtelomere were measured by qPCR and are normalized to IgG control, total histone H3, and input (see Materials and Methods). WT and *dot1Δ* (n = 1); *tlc1Δ* uninduced and induced (n = 2 each). (B) Anti-TERRA does not delay senescence when the N-terminus of Dot1 is absent. Senescence assays were performed using *dot1Δ* (n = 2) and *tlc1Δ dot1Δ* (n = 4) cells expressing the plasmid-borne Dot1^172-582^ C-terminal fragment and with anti-TERRA either induced or uninduced. (C) Anti-TERRA delays senescence by 11 PD (p = 0.0012) when the C-terminus of Dot1 is absent. Senescence assays were performed with anti-TERRA either induced or uninduced in *dot1Δ* (n = 2) and *tlc1Δ dot1Δ* (n = 5) cells expressing the plasmid-borne Dot1^1-237^ N-terminal fragment. For (B) and (C) each data point represents the mean PD versus the mean and SEM of the cell density.

The level of histone H3K79 methylation influences subtelomeric silencing [51,62–64] and is mainly found in transcriptionally active regions of the genome [58,59,63]. Thus, changes in H3K79 methylation might impact senescence. We performed chromatin immunoprecipitation (ChIP) for H3K79 trimethylation (H3K79me3) on senescent *tlc1Δ* samples induced or uninduced for anti-TERRA to determine whether anti-TERRA expression alters the ability of Dot1 to methylate the subtelomere. Although H3K79me3 levels increased several fold at senescence, anti-TERRA expression did not prevent this increase (Fig 5A). Therefore, anti-TERRA does not delay senescence by inhibiting H3K79me3 levels at subtelomeres.

Work from the VanLeeuwen laboratory has shown that the known functions of Dot1 can be genetically separated from one another [32]. The methyltransferase domain is located in the C-terminus of the protein [65] and a truncated protein containing only this portion of the protein (residues 172–582) is sufficient for normal levels of H3K79 methylation and the checkpoint activity of Dot1 [51,65,66]. In contrast, the Dot1 N-terminus (residues 1–237) cannot methylate H3K79 but is necessary and sufficient for Dot1-facilitated release of telomeres from the nuclear periphery [32]. We performed two quantitative liquid senescence assays where either of these N-terminal or C-terminal fragments were the only version of Dot1 present in the cell and where anti-TERRA was either induced or uninduced. Just as in a *tlc1Δ dot1Δ* strain, anti-TERRA expression did not delay senescence in the presence of only the C-terminal Dot1^172-582^ fragment (Fig 5B). This is consistent with our ChIP results (Fig 5A) and indicates that H3K79 methylation by Dot1 is not required for anti-TERRA to delay senescence. This is also consistent with its independence from Rad9 function since Dot1 checkpoint signaling through Rad9 requires H3K79me3 [52]. In contrast, the N-terminal portion of Dot1 (1–237) conferred a significant senescence delay upon anti-TERRA induction (p = 0.0012, Fig 5C). Thus, the N-terminus of Dot1 is both necessary and sufficient for senescence delay by anti-TERRA.

## Discussion

Here we show that expression of an anti-TERRA RNA, which binds native TERRA, significantly delays the senescence of yeast lacking telomerase. We also demonstrate that TERRA associates physically with Dot1 *in vivo* and *in vitro*, and that this interaction is inhibited by anti-TERRA expression. Although we cannot rule out the possibility that anti-TERRA has effects independent of TERRA, these findings together with the observation that deletion of *DOT1* delays senescence in a fashion epistatic to the delay provided by anti-TERRA, strongly suggest that anti-TERRA delays senescence by blocking cooperation between Dot1 and TERRA.

Previous work has also suggested that increased TERRA transcription drives senescence, but this idea has been based on non-physiological expression of TERRA-like molecules. Natural TERRA levels are extremely low, and in these earlier experiments, transcripts from a single telomere end were increased to levels >200 fold by integrating the strong and inducible *GAL1* promoter in subtelomeric sequences [49,67]. It is well documented that forcing such high levels of transcription through the telomeric repeats drives telomere shortening in *cis*, even in the presence of telomerase [68,69], and so it has been unclear whether natural levels of TERRA expression might have similar effects. Our new findings indicate that natural levels of TERRA, which are increased with telomere shortening, do indeed drive senescence.

Several mechanisms have been found to impact the rate of senescence in telomerase-deficient yeast, and we investigated whether they are related to the anti-TERRA mode of senescence delay. Of particular interest, it was shown that genetic inactivation of both RNase H activities in yeast (*rnh1Δ rnh201Δ*) delays senescence in a HR-dependent fashion that correlates with enhanced levels of RNA-DNA hybrids between TERRA and telomeric DNA [40,45,46]. Thus, it was argued that TERRA may delay senescence *via* HR-based telomere lengthening following the stalling of telomeric replication forks by R-loops formed between TERRA and telomeric DNA. This conclusion contrasts with the evidence that TERRA drives senescence, discussed above, and our new findings using anti-TERRA. Although it is plausible that the senescence delay afforded by RNase H-deficiency is explained by persistent TERRA-telomere R-loops, the delay cannot be attributed to these with certainty because telomeres compose less than 0.1 percent of the yeast genome, and thus the effects of RNase H deficiency in other genomic regions may be of importance. We note also that, in principle, anti-TERRA might impact levels of TERRA R-loops, e.g. destabilizing them by competing for base pairing between TERRA and telomere DNA or stabilizing them by base pairing with the displaced G-rich strand. However, anti-TERRA apparently delays senescence in a fashion distinct from these mechanisms because its mode of delay is unaffected by the absence or presence of *RAD52*, *RNH1*, and *RNH201*. Overall, because none of the approaches thus far taken to manipulate TERRA are known to impact TERRA selectively, more work is required to establish TERRA functions during yeast senescence.

Senescence can also be affected by the interplay of telomere shortening and the activities of epigenetic regulators [70–72]. For example, Dot1-mediated methylation of H3K79 has been thought to cooperate with Sas2 to enforce the boundary between heterochromatin and subtelomeric euchromatin [73–75]. Thus, we previously postulated that the senescence delays conferred by deletions of *DOT1* and *SAS2* are mechanistically similar [34]. In our current work, we detected increased subtelomeric levels of H3K79me3 at senescence, similar to the increased levels of H4K16 acetylation observed by Kozak et al. [34] Because these marks are associated with open chromatin, their elevated levels at subtelomeres are consistent with the observed increase in native TERRA levels at senescence. However, recent work demonstrating that H3K79me does not reduce Sir complex binding [63] and that loss of Dot1 does not affect Sir2 and Sir3 binding to subtelomeres [64] calls into question the antagonistic role of Sas2 and Dot1 in telomeric silencing. And moreover, we found that the delay in senescence provided by anti-TERRA depends on inhibition of the N-terminus of Dot1 and not the methylation activity provided by its C-terminus. That distinct mechanisms underlie the senescence delays provided by anti-TERRA/*dot1Δ* versus *sas2Δ* is also supported by differences in their requirement for HR and the non-epistatic relationship of anti-TERRA and *sas2Δ* (Fig 4 and S4 Fig, [34]). Thus, despite potentially collaborative roles in chromatin regulation, the functions of TERRA/Dot1 and Sas2 during senescence appear to be distinct.

Since the anti-TERRA senescence delay requires the N-terminus of Dot1, which can help release telomeres from the nuclear periphery [32], we hypothesize that such release may drive senescence. Tethering DNA to the nuclear periphery is a well-documented genome stabilizing mechanism. Loss of rDNA repeat anchoring to the periphery results in increased recombination and destabilization of the repeats [76]. Similarly, persistent double strand breaks (DSBs) are relocalized to the periphery and away from the rest of the genome thereby preventing inappropriate recombination events [77,78]. Telomeric tethering protects against aberrant recombination as loss of telomeric anchoring in S-phase results in Y’-specific amplification and a senescence-like phenotype in *tel1Δ* cells [79]. Intriguingly, persistent DSBs and critically shortened telomeres can also be localized to the nuclear pore complex (NPC; [80,81]). This has led to a model where telomeres (and persistent breaks) are first bound to the nuclear periphery by Mps3 and then shuttled to the NPC where repair may occur *via* the Slx5-Slx8 SUMO-dependent ubiquitin ligase complex [77,78,80,81].

As described above, telomeres are usually anchored to the nuclear periphery [79,82]. As telomeres become shorter during senescence, more TERRA is produced (Fig 1A and [23]) and these TERRA molecules can interact with Dot1 (Fig 4). We speculate that this may cause release of the critically shortened telomeres from their normal sites of peripheral anchoring and thus enable relocalization of telomeres to NPCs where they may signal cellular senescence [80]. If anti-TERRA is expressed, Dot1 is no longer able to interact with TERRA, delaying senescence. Perhaps because TERRA molecules contain subtelomeric portions unique to the telomere from which they were transcribed, they could allow for recruitment of Dot1 back to the telomere of origin, consistent with evidence that the shortest telomere signals senescence [83]. Thus, similar to the idea that TERRA can recruit telomerase to the shortest telomere [23], we suggest that if TERRA cannot recruit telomerase, as would be the case in telomerase-deficient cells, it may instead recruit Dot1 which aids in signaling cellular senescence by releasing telomeres from the nuclear periphery.

## Supporting information

S1 Text Supplemental materials and methods. (PDF)

S1 Fig MS2-tagged anti-TERRA causes a similar senescence delay to untagged anti-TERRA upon induction. MS2-tagged anti-TERRA expression delays senescence in *tlc1* mutants by 10 PD (p < 0.0001), which is the same as for non-MS2-tagged anti-TERRA. *TLC1/tlc1Δ* diploids were sporulated and senescence assays of WT (n = 2) and *tlc1Δ* (n = 5) haploids were performed as indicated in the *Materials and Methods* with MS2-tagged anti-TERRA either induced or uninduced. Each data point represents the mean PD versus the mean and SEM of the cell density.
(TIFF)

S2 Fig Anti-TERRA causes a senescence delay in *est2Δ* cells. Induction of anti-TERRA delays senescence by 10 PD in an *est2Δ tlc1Δ* background (p = 0.016). *EST2/est2Δ* diploids were sporulated and senescence assays of WT (n = 2) and *est2Δ* (n = 5) were performed as indicated in the *Materials and Methods* with anti-TERRA either induced or uninduced without pre-incubation in raffinose. Each data point represents the mean PD versus the mean and SEM of the cell density.
(TIFF)

S3 Fig Anti-TERRA does not generate novel sequences at the telomeres of senescing cells. Genomic DNA was isolated from *tlc1Δ rad52Δ* haploids bearing the anti-TERRA plasmid and which had senesced under uninduced or induced conditions for the indicated population doubling (PD) after spore germination. *RAD52* was deleted to avoid recombination-dependent events that could have generated novel telomere sequences. Chromosome 1L telomeres were tailed, PCR amplified, cloned and sequenced as described in S1 Text. Telomeres are sorted by length with internal sequence differences indicated by a lack of gray highlighting and unique sequence at the 3’ terminus identified by bold text and yellow highlight. There is no difference between induced and uninduced conditions in the number of telomeres with unique sequence at the termini (two-tailed Fisher’s exact test, p = 0.7).
(TIFF)

S4 Fig The anti-TERRA senescence delay is not dependent on Tel1, Sas2, RNase H enzymes, or Rad9. (A and B) Anti-TERRA delays senescence in *tlc1Δ tel1Δ* mutants and *tlc1Δ sas2Δ* mutants. *TLC1/tlc1Δ TEL1/tel1Δ SAS2/sas2Δ RAD52/rad52Δ* diploids were sporulated and senescence assays of *tlc1Δ* (n = 3), *tlc1Δ tel1Δ* (n = 4) and *tlc1Δ sas2Δ* (n = 4) were performed with anti-TERRA either induced or uninduced as indicated in the *Materials and Methods* without pre-incubation in raffinose. The *tlc1Δ* data is the same in both panels since all assays are from the same diploid strain and were performed at the same time. (A) Anti-TERRA delays senescence an additional 13 PD more than *tel1Δ* alone (p = 0.0007, uninduced *tlc1Δ tel1Δ* versus induced). (B) Anti-TERRA delays senescence 10 PD more than *sas2Δ* alone (p = 0.0002, uninduced *tlc1Δ sas2Δ* versus induced). (C) Anti-TERRA delays senescence in *rnh1Δ rnh201Δ tlc1Δ* mutants by an additional 12 PD (p = 0.002). *TLC1/tlc1Δ RNH1/rnh1Δ RNH201/rnh201Δ RAD52/rad52Δ* diploids were sporulated and senescence assays of *rnh1Δ rnh201Δ* (n = 2) and *tlc1Δ rnh1Δ rnh201Δ* (n = 5) were performed as indicated in the *Materials and Methods* with anti-TERRA either induced or uninduced. (D) Anti-TERRA expression delays senescence in *rad9Δ tlc1Δ* mutants by 9 PD (p = 0.02). *TLC1/tlc1Δ RAD9/rad9Δ* diploids were sporulated and senescence assays of *rad9Δ* (n = 2) and *tlc1Δ rad9Δ* (n = 5) were performed as indicated in the *Materials and Methods* with anti-TERRA either induced or uninduced. In our hands, *rad9Δ tlc1Δ* did not show a senescence delay versus *tlc1Δ* alone [84,85]. For all panels, each data point represents the mean PD versus the mean and SEM of the cell density.
(TIFF)

S5 Fig Anti-TERRA expression does not rescue the temperature sensitivity of *yku70Δ* or *yku80Δ* mutants. Strains bearing the control or anti-TERRA plasmid were grown under conditions both selecting for the plasmid and inducing its expression during the entire assay. Strains were serially diluted, plated, and grown for 2 to 3 days at the temperatures indicated as described in S1 Text.
(TIFF)

S1 Table Strains used in this study. (PDF)

S2 Table Oligonucleotides used in this study for qPCR. (PDF)
