# Supplementary material for: TERRA and the histone methyltransferase Dot1 cooperate to regulate senescence in budding yeast
Source: PLoS One. 2018 Apr 12;13(4):e0195698. doi: 10.1371/journal.pone.0195698 (PMC5896980; doi:10.1371/journal.pone.0195698)
Supplement: S1 Text — (PDF) [file pone.0195698.s001.pdf]

Spot assays

Haploid colonies containing either pGL2 or pGL3 were grown in SC-HIS+2% galactose on a roller drum at room temperature (23°C) until they reached log phase growth, counted with a Coulter counter, and diluted in sterile water to 10^5 cells/15 μL. 10-fold serial dilutions (10^5 to 10^1) were performed and about 15 μL of each dilution was stamped onto SC-HIS+galactose plates with a pinner and incubated at either room temperature or 37°C for 2-3 days.

Telomere PCR and sequencing

Telomere PCR was performed as described in [1] except after denaturation and C-tailing, the telomere ends were amplified using Accuprime GC-rich DNA polymerase (Invitrogen), Buffer A (comes with Accuprime), and a 3mM final concentration of magnesium sulfate. Then, 0.7U of Taq DNA polymerase was added to each reaction and incubated at 72°C for 8-10 minutes before purification and cloning. Sequences were aligned using VectorNTI.

References

1. Kozak ML, Chavez A, Dang W, Berger SL, Ashok A, Guo X, et al. Inactivation of the Sas2 histone acetyltransferase delays senescence driven by telomere dysfunction. EMBO J. 2010;29: 158–70. doi:10.1038/emboj.2009.314
