# Supplementary figures and images for: TERRA and the histone methyltransferase Dot1 cooperate to regulate senescence in budding yeast
Source: PLoS One. 2018 Apr 12;13(4):e0195698. doi: 10.1371/journal.pone.0195698 (PMC5896980; doi:10.1371/journal.pone.0195698)

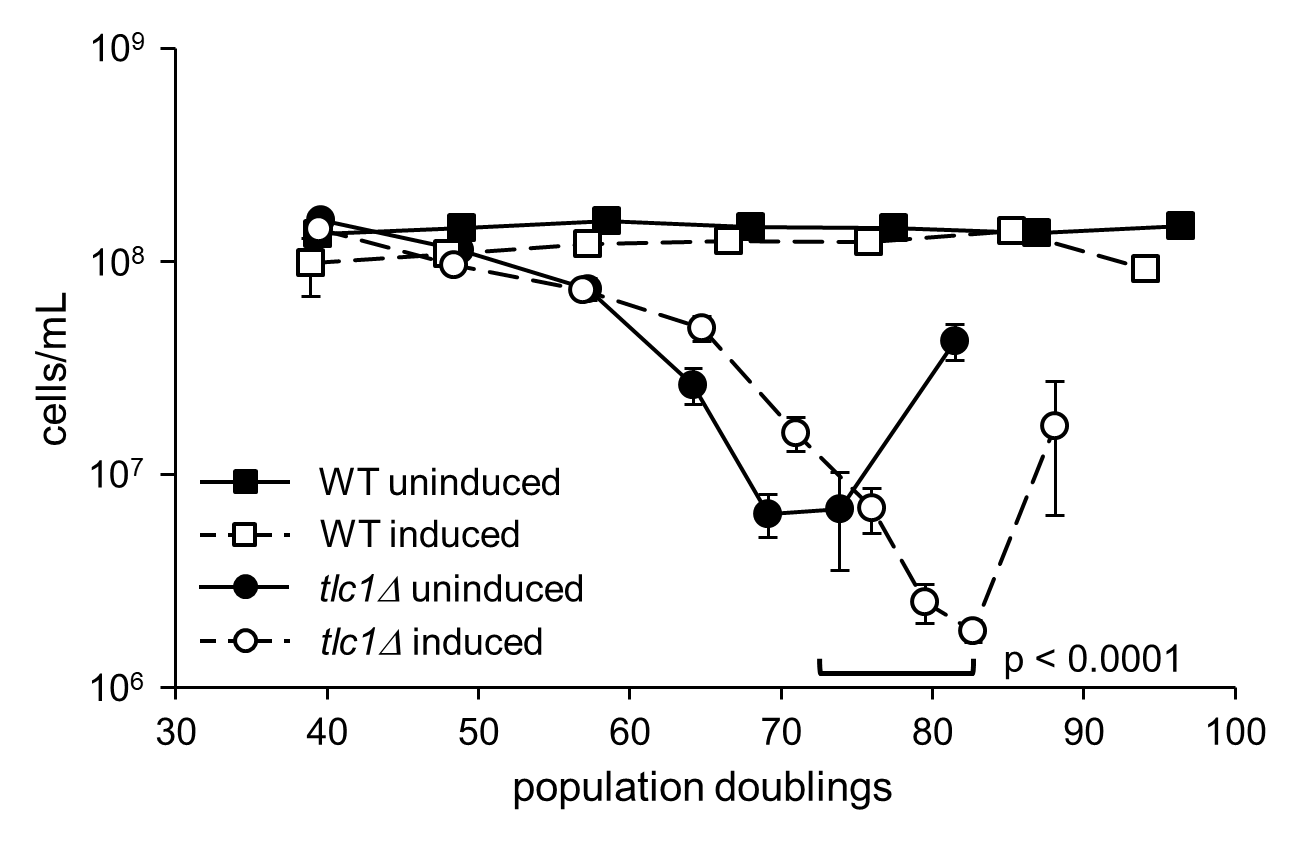

Supplement: S1 Fig — MS2-tagged anti-TERRA expression delays senescence in tlc1 mutants by 10 PD (p < 0.0001), which is the same as for non-MS2-tagged anti-TERRA. TLC1/tlc1Δ diploids were sporulated and senescence assays of WT (n = 2) and tlc1Δ (n = 5) haploids were performed as indicated in the Materials and Methods with MS2-tagged anti-TERRA either induced or uninduced. Each data point represents the mean PD versus the mean and SEM of the cell density. (TIFF) [file pone.0195698.s002.tiff]

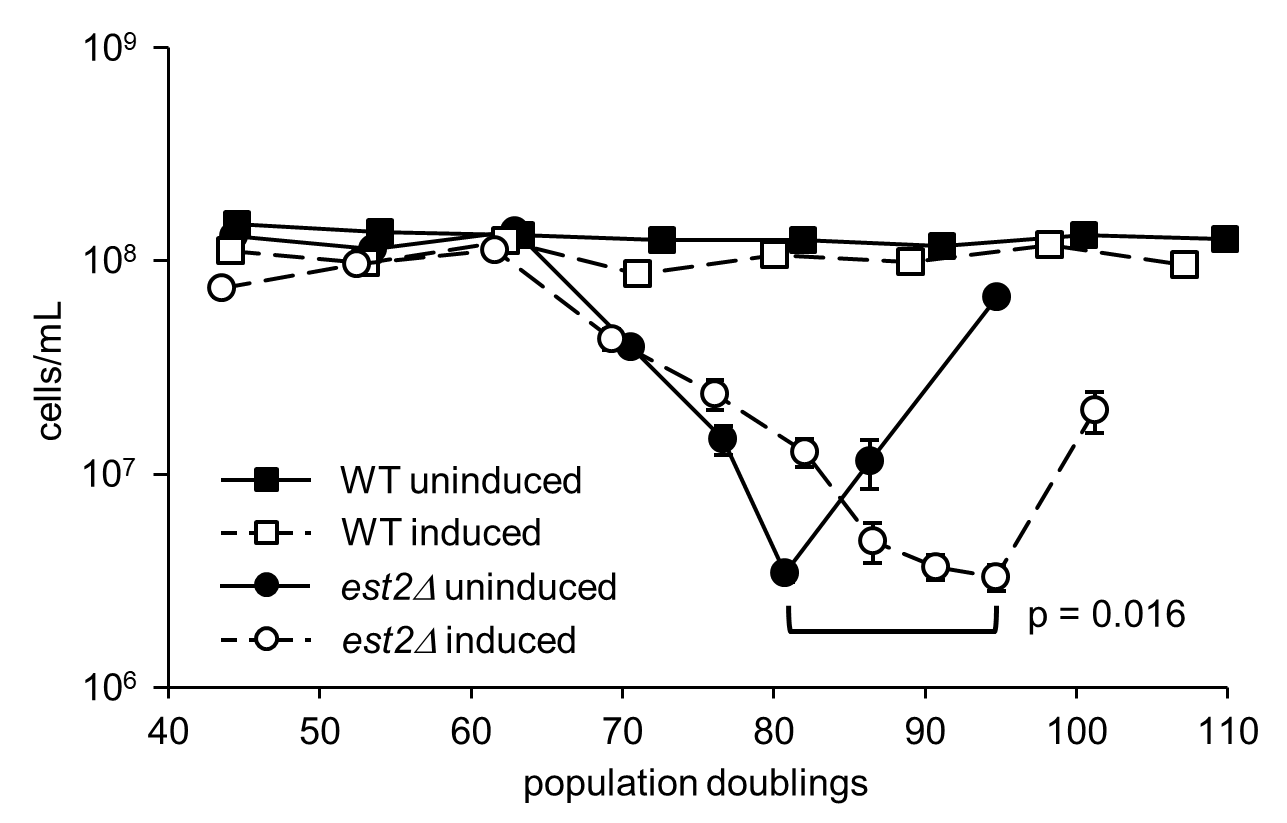

Supplement: S2 Fig — Induction of anti-TERRA delays senescence by 10 PD in an est2Δ tlc1Δ background (p = 0.016). EST2/est2Δ diploids were sporulated and senescence assays of WT (n = 2) and est2Δ (n = 5) were performed as indicated in the Materials and Methods with anti-TERRA either induced or uninduced without pre-incubation in raffinose. Each data point represents the mean PD versus the mean and SEM of the cell density. (TIFF) [file pone.0195698.s003.tiff]

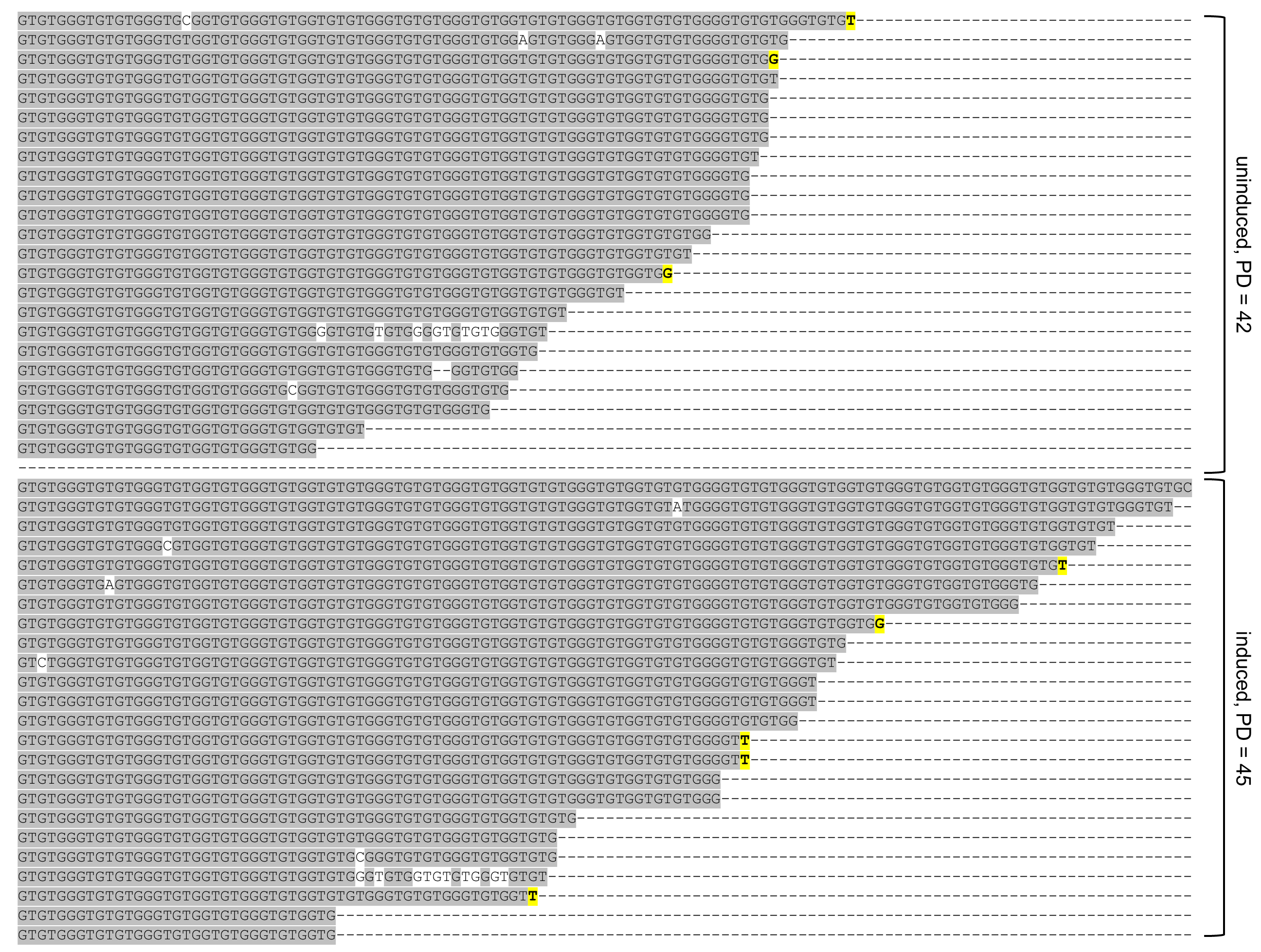

Supplement: S3 Fig — Genomic DNA was isolated from tlc1Δ rad52Δ haploids bearing the anti-TERRA plasmid and which had senesced under uninduced or induced conditions for the indicated population doubling (PD) after spore germination. RAD52 was deleted to avoid recombination-dependent events that could have generated novel telomere sequences. Chromosome 1L telomeres were tailed, PCR amplified, cloned and sequenced as described in S1 Text. Telomeres are sorted by length with internal sequence differences indicated by a lack of gray highlighting and unique sequence at the 3’ terminus identified by bold text and yellow highlight. There is no difference between induced and uninduced conditions in the number of telomeres with unique sequence at the termini (two-tailed Fisher’s exact test, p = 0.7). (TIFF) [file pone.0195698.s004.tiff]

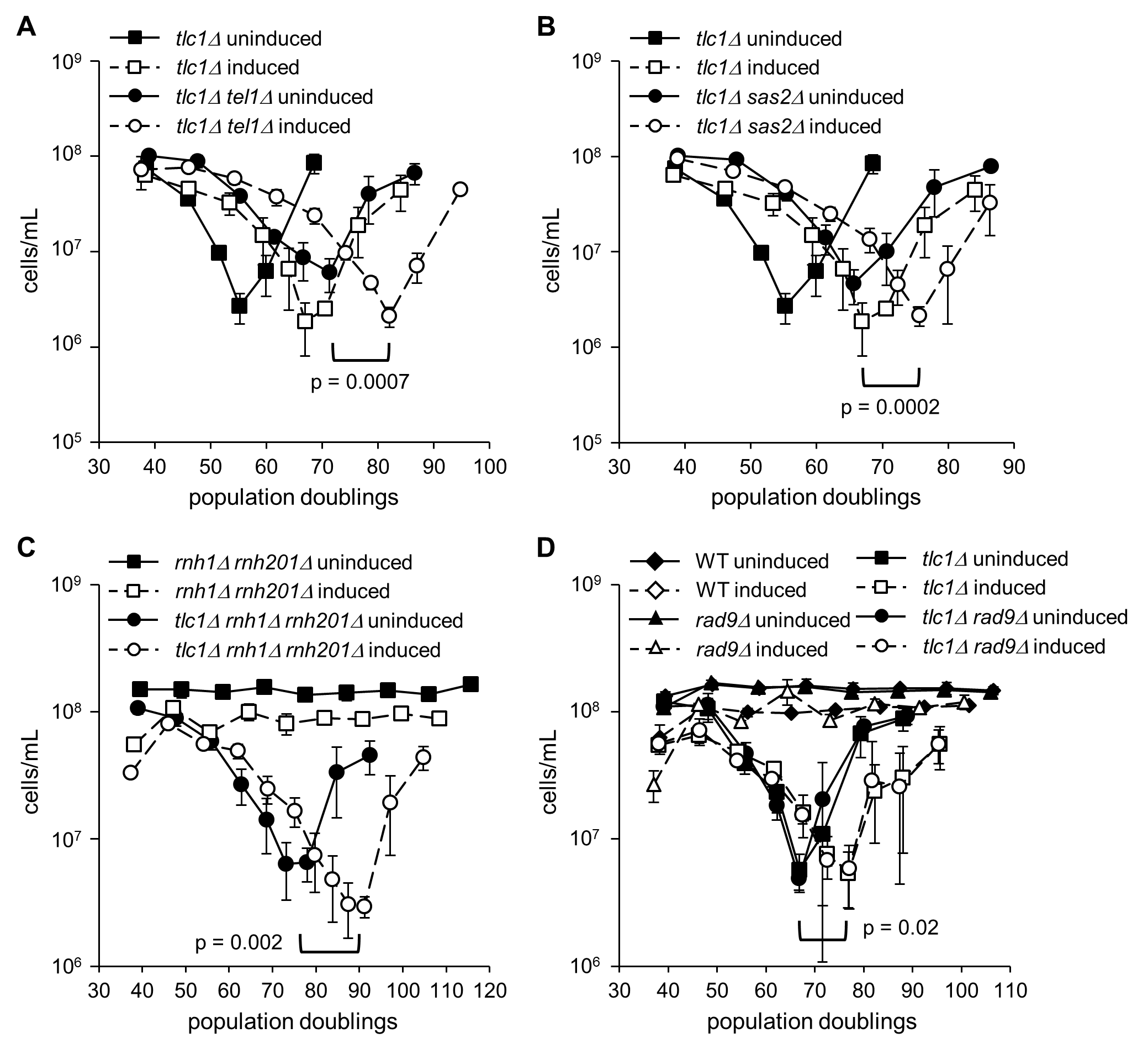

Supplement: S4 Fig — (A and B) Anti-TERRA delays senescence in tlc1Δ tel1Δ mutants and tlc1Δ sas2Δ mutants. TLC1/tlc1Δ TEL1/tel1Δ SAS2/sas2Δ RAD52/rad52Δ diploids were sporulated and senescence assays of tlc1Δ (n = 3), tlc1Δ tel1Δ (n = 4) and tlc1Δ sas2Δ (n = 4) were performed with anti-TERRA either induced or uninduced as indicated in the Materials and Methods without pre-incubation in raffinose. The tlc1Δ data is the same in both panels since all assays are from the same diploid strain and were performed at the same time. (A) Anti-TERRA delays senescence an additional 13 PD more than tel1Δ alone (p = 0.0007, uninduced tlc1Δ tel1Δ versus induced). (B) Anti-TERRA delays senescence 10 PD more than sas2Δ alone (p = 0.0002, uninduced tlc1Δ sas2Δ versus induced). (C) Anti-TERRA delays senescence in rnh1Δ rnh201Δ tlc1Δ mutants by an additional 12 PD (p = 0.002). TLC1/tlc1Δ RNH1/rnh1Δ RNH201/rnh201Δ RAD52/rad52Δ diploids were sporulated and senescence assays of rnh1Δ rnh201Δ (n = 2) and tlc1Δ rnh1Δ rnh201Δ (n = 5) were performed as indicated in the Materials and Methods with anti-TERRA either induced or uninduced. (D) Anti-TERRA expression delays senescence in rad9Δ tlc1Δ mutants by 9 PD (p = 0.02). TLC1/tlc1Δ RAD9/rad9Δ diploids were sporulated and senescence assays of rad9Δ (n = 2) and tlc1Δ rad9Δ (n = 5) were performed as indicated in the Materials and Methods with anti-TERRA either induced or uninduced. In our hands, rad9Δ tlc1Δ did not show a senescence delay versus tlc1Δ alone [84,85]. For all panels, each data point represents the mean PD versus the mean and SEM of the cell density. (TIFF) [file pone.0195698.s005.tiff]

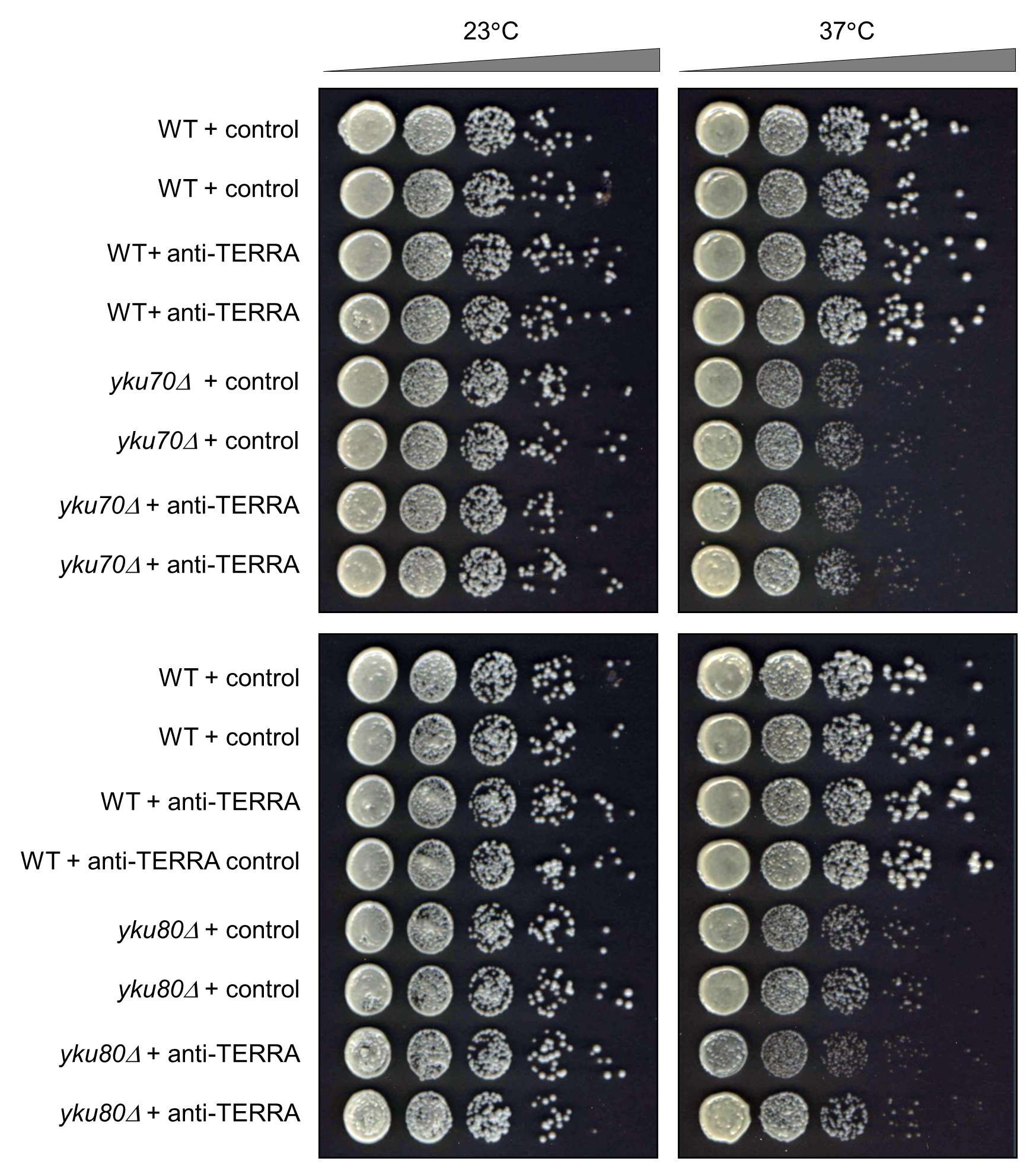

Supplement: S5 Fig — Strains bearing the control or anti-TERRA plasmid were grown under conditions both selecting for the plasmid and inducing its expression during the entire assay. Strains were serially diluted, plated, and grown for 2 to 3 days at the temperatures indicated as described in S1 Text. (TIFF) [file pone.0195698.s006.tiff]
