# Supplementary material for: TERRA and the histone methyltransferase Dot1 cooperate to regulate senescence in budding yeast
Source: PLoS One. 2018 Apr 12;13(4):e0195698. doi: 10.1371/journal.pone.0195698 (PMC5896980; doi:10.1371/journal.pone.0195698)
Supplement: S1 Table — (PDF) [file pone.0195698.s007.pdf]

Strain	Genotype	Source
YJW5	*Mata/α his3Δ1/his3Δ1 leu2Δ0/leu2Δ0 ura3Δ0/ura3Δ0 MET15/met15Δ0 LYS2/lys2Δ0*	[1]
YJW25	YJW5 + *EST2/est2Δ::HygMX4* + pGL3	This study.
YJW26	YJW5 + *EST2/est2Δ::HygMX4* + pGL2	This study.
YJW6	YJW5 + *TLC1/tlc1Δ::LEU2*	This study.
YGL2	YJW6 + pGL2	This study.
YGL3	YJW6 + pGL3	This study.
YJW170	YJW6 + pJD1	This study.
YJW31	*Mata/α TLC1/tlc1Δ::LEU2 EXO1/exo1Δ::CaURA3 his3Δ1/his3Δ1 leu2Δ0/leu2Δ0 ura3Δ0/ura3Δ0 MET15/met15Δ0 LYS2/lys2Δ0* + pGL2	This study.
YJW198	*Mata/α TLC1/tlc1Δ::LEU2 DOT1/dot1Δ::KanMX4 his3Δ1/his3Δ1 leu2Δ0/leu2Δ0 ura3Δ0/ura3Δ0 MET15/met15Δ0 LYS2/lys2Δ0* + pGL2	This study.
YJW287	*Mata/α TLC1/tlc1Δ::HygMX4 DOT1/dot1Δ::KanMX4 his3Δ1/his3Δ1 leu2Δ0/leu2Δ0 ura3Δ0/ura3Δ0 met15Δ0/met15Δ0 LYS2/LYS2*	This study.
YJW300	YJW287 + pFvL901 + pGL2	This study.
YJW301	YJW287 + pFvL905 + pGL2	This study.
YJW303	YJW287 + pFvL914 + pGL2	This study.

YJW291	*Mata/α RAD9/rad9Δ::KanMX4 TLC1/tlc1Δ::LEU2 his3Δ1/his3Δ1 leu2Δ0/leu2Δ0 ura3Δ0/ura3Δ0 met15Δ0/met15Δ0 LYS2/LYS2* + pGL2	This study.
YJW292	*Mata/α TLC1/tlc1Δ::LEU2 Rad52/rad52Δ::HygMX4 RNH1/rnh1Δ::KanMX4 RNH201/rnh201Δ::KanMX4 his3Δ1/his3Δ1 leu2Δ0/leu2Δ0 ura3Δ0/ura3Δ0 MET15/met15Δ0 LYS2/lys2Δ0* + pGL2	This study.
YJW417	*Mata/α RAD52/rad52::HygMX4 TLC1/tlc1Δ::HygMX4 DOT1/dot1Δ::KanMX4 his3Δ1/his3Δ1 leu2Δ0/leu2Δ0 ura3Δ0/ura3Δ0 MET15/met15Δ0 lys2Δ0/lys2Δ0* + pGL2	This study.
YMK324	*Mata/α TLC1/tlc1Δ::LEU2 TEL1/tel1Δ::KanMX4 SAS2/sas2Δ::HygMX4 his3Δ1/his3Δ1 leu2Δ0/leu2Δ0 ura3Δ0/ura3Δ0 MET15/met15Δ0 LYS2/lys2Δ0*	[2]
YGL17	YMK324 + pGL2	This study.
YGL18	YMK324 + pGL3	This study.
YJW40	*Mata his3Δ1 leu2Δ0 met15Δ0 ura3Δ0* + pGL2	This study.
YJW41	Biological replicate of YJW40.	This study.
YJW48	*Mata yKu70Δ::KanMX4 his3Δ1 leu2Δ0 met15Δ0 ura3Δ0* + pGL2	This study.
YJW49	Biological replicate of YJW48.	This study.
YJW50	*Mata yKu80Δ::KanMX4 his3Δ1 leu2Δ0 met15Δ0 ura3Δ0* + pGL2	This study.

YJW51	Biological replicate of YJW50.	This study.
YJW66	*Mata yKu70Δ::KanMX4 his3Δ1 leu2Δ0 met15Δ0 ura3Δ0* + pGL3	This study.
YJW67	Biological replicate of YJW66.	This study.
YJW68	*Mata yKu80Δ::KanMX4 his3Δ1 leu2Δ0 met15Δ0 ura3Δ0* + pGL3	This study.
YJW69	Biological replicate of YJW68.	This study.
YJW78	*Mata his3Δ1 leu2Δ0 met15Δ0 ura3Δ0* + pGL3	This study.
YJW79	Biological replicate of YJW78.	This study.

References

1. Brachmann CB, Davies a, Cost GJ, Caputo E, Li J, Hieter P, et al. Designer deletion strains derived from Saccharomyces cerevisiae S288C: a useful set of strains and plasmids for PCR-mediated gene disruption and other applications. Yeast. 1998;14: 115–32. doi:10.1002/(SICI)1097-0061(19980130)14:2<115::AID-YEA204>3.0.CO;2-2
2. Kozak ML, Chavez A, Dang W, Berger SL, Ashok A, Guo X, et al. Inactivation of the Sas2 histone acetyltransferase delays senescence driven by telomere dysfunction. EMBO J. 2010;29: 158–70. doi:10.1038/emboj.2009.314
