# Supplementary material for: TERRA and the histone methyltransferase Dot1 cooperate to regulate senescence in budding yeast
Source: PLoS One. 2018 Apr 12;13(4):e0195698. doi: 10.1371/journal.pone.0195698 (PMC5896980; doi:10.1371/journal.pone.0195698)
Supplement: S2 Table — (PDF) [file pone.0195698.s008.pdf]

Oligonucleotide	Sequence	Reference
SPC42 F	AAGAGCTGCAAAGCATGATGGAC	[1]
SPC42 R	GACTGGATTGGGAAGAATGACGA	[1]
10R F	CGGTTATGGTGGACGGTGGATG	[2]
10R R	CCTAACCCTATTCTAATCCAACCCTGATAA	[2]
13R F	ACGGTTATGGTGCACGATGGG	[2]
13R R	TTACCCTCCATTACGCTACCTCC	[2]
15L F	GGGTAACGAGTGGGGAGGTAA	[2]
15L R	CAACACTACCCTAATCTAACCCTGT	[2]
6R F	GGCAAGGGTAAAAACCAGTGAGG	This study.
6R R	GACCCAGTCCTCATTTCCATCAA	This study.
4L F	GGAGTGGATGGTTGAGTGGGG	[2]
4L R	CTAACACTACCCTATTCTAACCCTGATTTT	[2]
10R/14R F	GGATGGTGGTTGGAGTTGTAGAATG	[2]
10R/14R R	ATCCAACCCTGATAAACCTGTCTCTT	[2]
1L F	TGGCCAACCTGTCTCTCAACTT	This study.
1L R	AGGGTAACGGTGGGTGAGTGGTA	This study.
Y'10 F	TTTGGTTGAACATCCGGGTAAGA	This study.
Y'10 R	CATAGCCCTAAATAGCCCTAAATAGCC	This study.
Y'6 F	GGCTTGGAGGAGACGTACATG	[2]
Y'6 R	CTCGCTGTCACTCCTTACCCG	[2]
Y'3 F	GGCTTGGAGGAGACGTACATG	[2]
Y'3 R	CCACACACTCTCTCACATCTACCTC	[2]

Tel6R-0.1-F	TGAGGCCATTTCCGTGTGTA	[3]
Tel6R-0.1-R	CCCAGTCCTCATTTCCATCAA	[3]
Tel6R-0.2-F	CCTTTTTTGATATAACTGTCGGAGAGT	[3]
Tel6R-0.2-R	TCCGAACGCTATTCCAGAAAGT	[3]
Tel6R-3.6-F	TCTCGGTGAACGGATGCA	[3]
Tel6R-3.6-R	CACGACAAAACCACCGATGA	[3]
Tel6R-7.3-F	TGCGAAATAAGAACACGATCGT	[3]
Tel6R-7.3-R	GTAGAAGGGCCGACATGTACTACA	[3]
CA F	ACACACCACACCACACACCACAC	This study.
CA R	GGCGTGAATGTAAGCGTGACATAA	This study.

References

1. Platt JM, Ryvkin P, Wanat JJ, Donahue G, Ricketts MD, Barrett SP, et al. Rap1 relocalization contributes to the chromatin-mediated gene expression profile and pace of cell senescence. Genes Dev. 2013;27: 1406–20. doi:10.1101/gad.218776.113
2. Iglesias N, Redon S, Pfeiffer V, Dees M, Lingner J, Luke B. Subtelomeric repetitive elements determine TERRA regulation by Rap1/Rif and Rap1/Sir complexes in yeast. EMBO Rep. 2011;12: 587–93. doi:10.1038/embor.2011.73
3. Kozak ML, Chavez A, Dang W, Berger SL, Ashok A, Guo X, et al. Inactivation of the Sas2 histone acetyltransferase delays senescence driven by telomere dysfunction. EMBO J. 2010;29: 158–70. doi:10.1038/emboj.2009.314
